# Phylogenetic Analyses of *Armillaria* Reveal at Least 15 Phylogenetic Lineages in China, Seven of Which Are Associated with Cultivated *Gastrodia elata*

**DOI:** 10.1371/journal.pone.0154794

**Published:** 2016-05-03

**Authors:** Ting Guo, Han Chen Wang, Wan Qiu Xue, Jun Zhao, Zhu L. Yang

**Affiliations:** 1 Key Laboratory for Plant Diversity and Biogeography of East Asia, Kunming Institute of Botany, Chinese Academy of Sciences, Heilongtan, Kunming 650201, China; 2 College of Life Sciences, Chongqing Normal University, Chongqing, 401331, China; 3 University of Chinese Academy of Sciences, No. 19A Yuquan Road, Beijing 100049, China; 4 General Station of Forest Pest Control, State Forestry Administration, Shenyang 110034, China; USDA Forest Service—RMRS, UNITED STATES

## Abstract

Fungal species of *Armillaria*, which can act as plant pathogens and/or symbionts of the Chinese traditional medicinal herb *Gastrodia elata* (“Tianma”), are ecologically and economically important and have consequently attracted the attention of mycologists. However, their taxonomy has been highly dependent on morphological characterization and mating tests. In this study, we phylogenetically analyzed Chinese *Armillaria* samples using the sequences of the internal transcribed spacer region, translation elongation factor-1 alpha gene and beta-tubulin gene. Our data revealed at least 15 phylogenetic lineages of *Armillaria* from China, of which seven were newly discovered and two were recorded from China for the first time. Fourteen Chinese biological species of *Armillaria*, which were previously defined based on mating tests, could be assigned to the 15 phylogenetic lineages identified herein. Seven of the 15 phylogenetic lineages were found to be disjunctively distributed in different continents of the Northern Hemisphere, while eight were revealed to be endemic to certain continents. In addition, we found that seven phylogenetic lineages of *Armillaria* were used for the cultivation of Tianma, only two of which had been recorded to be associated with Tianma previously. We also illustrated that *G*. *elata* f. *glauca* (“Brown Tianma”) and *G*. *elata* f. *elata* (“Red Tianma”), two cultivars of Tianma grown in different regions of China, form symbiotic relationships with different phylogenetic lineages of *Armillaria*. These findings should aid the development of Tianma cultivation in China.

## Introduction

Fungal species of the genus *Armillaria* (Fr.: Fr.) Staude (Physalacriaceae, Agaricales, Basidiomycota) are well known as important plant pathogens that can cause serious root diseases on diverse trees and woody plants growing in plantations, forests and orchards worldwide, resulting in huge economic losses [1, 2]. However, significant differences in pathogenicity have been observed among species of *Armillaria*. For example, *A*. *ostoyae* (Romagn.) Herink (= *A*. *solidipes* [3, 4]) and *A*. *mellea* (Vahl) P. Kumm. are generally considered to be strongly pathogenic, whereas species such as *A*. *sinapina* Bérubé & Dessur., *A*. *gallica* Marxm. & Romagn. and *A*. *cepistipes* Velen. are considered less pathogenic [2]. Therefore, accurate species delimitation of *Armillaria* is critical for the assessment of disease risk.

Several species of *Armillaria* have been identified as symbionts of *Gastrodia elata* Bl. (“Tianma” in Chinese) and *Polyporus umbellatus* (Pers.) Fr., both being well known as important elements of traditional medicine in East Asia [5–8]. Based on data from the Ministry of Commerce, the output of fresh tubers of Tianma reached a record 14,873 tonnes in 2015, generating an annual production value up to $100 million USD (http://www.gov.cn/xinwen/2015-07/22/content_2900532.htm). The growth and maturation of Tianma, a heterotrophic perennial orchid with no leaves and roots, heavily rely on the presence of *Armillaria* partners [9]. It is well known that different strains of *Armillaria* can affect both the production and the quality of the tubers of Tianma [9, 10]. Thus, screening the best *Armillaria* strains and assigning them to the correct taxa are particularly important for the cultivation of Tianma.

In the past 40 years, mating tests have been widely used in the taxonomy of *Armillaria*. Using these tests, 16 Chinese Biological species (CBS A to CBS P) of *Armillaria* have been identified [11, 12]. However, mating tests rely on the availability of single-spore (haploid) isolates. This limits their use in the identification of samples collected as rhizomorphs, which are commonly associated with Tianma tubers. Furthermore, the results obtained from mating tests are sometimes ambiguous and inconclusive. The mating results are judged by the change in colony morphology. The reaction tends to be indistinct if the mating tests are performed on diploid–haploid or diploid–diploid pairings [12–14]. For example, in some pairings between Japanese homothallic isolates, unclear zone lines were observed, and the scenario also occurred that there was no pigmented line at the junction of the two isolates but the mycelia did not intermingle on either side [15].

In recent years, molecular data, specifically DNA sequence data, have been increasingly used to identify fungal species [16]. Taylor et al. [17] introduced the Genealogical Concordance Phylogenetic Species Recognition (GCPSR) as an objective way to define the limits of sexual species. Since their publication, numerous complexes of sibling species have been uncovered in fungi using the multilocus approach [18, 19]. Tsykun et al. [20] used a multilocus approach for the identification of *Armillaria* species occurring in Europe. Three loci, the rDNA ITS, IGS-1 (intergenic spacer region one) and the translation elongation factor-1 alpha (*tef1*-*α*), were used in their study. Among these, it was shown that *tef1*-*α* could successfully distinguish closely related *Armillaria* biological species, which has also been revealed by other researchers [21–25]. Based on the sequences from *tef1*-*α* and IGS-1, a recent study by Coetzee et al. [26] elucidated the phylogenetic relationships among biological species of *Armillaria* from China, and generally resolved four main phylogenetic groups, namely, the “*A*. *ostoyae*”, “*A*. *gallica*”, “*A*. *tabescens*” and “*A*. *mellea*” clusters. However, the relationship between the CBS and the phylogenetic clades is still unresolved, with most CBS (i.e., CBS C, F, G, H, J, L, N and O) remaining unnamed.

In this study, we extensively collect samples of *Armillaria* from China and analyze them using sequences of three DNA regions. Together with the rDNA ITS and *tef1*-*α*, which have been used in studies of *Armillaria* before, sequences from the beta-tubulin (*β*-*tubulin*) gene are also used, as it has been demonstrated that the third codons and introns of *β*-*tubulin* are highly variable and thus can be used at the interspecific level and even at the intraspecific level in fungal taxonomy [27–29]. In this study, we aim to i) reveal the phylogenetic diversity of *Armillaria* in China and establish the link between the CBS and the phylogenetic lineages; ii) determine the phylogenetic relationships among *Armillaria* samples from different regions of the Northern Hemisphere; and iii) assess the diversity of the phylogenetic lineages of *Armillaria* associated with Tianma in China.

## Materials and Methods

### Ethics statement

*Armillaria* is neither protected nor endangered in the sampled areas, and all samples were collected by researchers following current Chinese regulations. None of the sampled locations are privately owned or protected by law.

### Taxon sampling, strain collection and isolation

*Armillaria* samples used in this study comprised three types: i) single-spore isolates representing 14 out of 16 known CBS (except CBS E and CBS P; the two isolates of CBS E were assigned to CBS C and CBS D by Zhao et al. [30] and the isolates of CBS P were not available), each with at least three single-spore isolates. They were all cultures previously assigned to biological species by one of the present authors (HCW) or other mycologists [11, 12, 31–37]; ii) basidiomata collected during the past 10 years, mainly from Southwest China; and iii) rhizomorphs/samples associated with Tianma, including rhizomorphs attached to the tuber surfaces, commercial strains of *Armillaria* used for the cultivation of Tianma, and rhizomorphs or basidiomata collected from inoculum (wood infected with *Armillaria* strains by farmers) on Tianma plantations in northeastern Yunnan during 2010–2014. Table 1 shows the details of all of the samples. Vouchers were kept in the Herbarium of Cryptogams of the Kunming Institute of Botany, Chinese Academy of Sciences (HKAS). For the rhizomorphs and some basidiomata, we prepared pure cultures to facilitate DNA isolation. All of the pure cultures were made from a variety of tissues following standard aseptic techniques. Pieces approximately 3–5 mm in length taken at the junction of the stipe and pileus of the basidiomata were inoculated on potato dextrose agar (PDA) media. Rhizomorphs were rinsed overnight with distilled water and sterilized with 75% ethanol for 1 min. The rhizomorphs were then carefully dried using sterilized filter paper, plated onto PDA media in Petri dishes, and cultured at 22–24°C in the dark. For the strains associated with Tianma, images of rhizomorphs were taken after about 2 weeks to compare the branching development. All of these isolates were preserved in the Key Laboratory for Plant Diversity and Biogeography of East Asia, Kunming Institute of Botany (KIB) of the Chinese Academy of Sciences.

**Table 1:** Collections of *Armillaria* used in this study.

Taxon	Biological	Herbarium ID	Isolated ID	Origin	Location	GenBank accession numbers		
	species ^1^					ITS	*tef1-α*	*β-tubulin*
*A*. *borealis*	*A*. *borealis*	HKAS 86616	97061/18^2^	tester strain	Helsinki, Finland	KT822291	KT822427	KT822214
*A*. *borealis*	/	HKAS 76263	Gt571	basidioma	Muli, SC	KT822294	KT822426	KT822193
*A*. *borealis*	/	HKAS 56108	Li1254	basidioma	Xiangshan, YN	KT822293	KT822425	-
*A*. *cepistipes*	/	HKAS 86542	SN2^d^	commercial strain	Yiliang, YN	KT822303	KT822421	KT822179
*A*. *cepistipes*	CBS F	HKAS 86583	01108/1^1^	single spore	Xinlin, HLJ	KT822290	KT822417	KT822181
*A*. *cepistipes*	CBS F	HKAS 86584	96059/86^1^	single spore	Laotuding, LN	KT822278	KT822419	KT822178
*A*. *cepistipes*	CBS F	HKAS 86585	96060/21^1^	single spore	Laotuding, LN	KT822283	KT822420	KT822180
*A*. *cepistipes*	CBS F	HKAS 86587	99101/7^1^	single spore	Yichun, HLJ	KT822287	KT822418	KT822182
*A*. cf. *borealis*	(CBS M) *A*. *borealis*	HKAS 86617	01015/7^1^	single spore	Shennongjia, HB	KT822309	KT822432	KT822218
*A*. cf. *gallica*	/	HKAS 85517	Guo408	basidioma	XJ	KT822312	KT822409	KU378051
*A*. *ectypa*		HKAS 86565	70011/13^2^	strain	Auvergne, France	KT822340	KT822438	KT822241
*A*. *gallica*	*A*. *gallica*	HKAS 86569	93421/1^2^	tester strain	EU	KT822277	KT822414	KT822203
*A*. *mellea*	/	HKAS 85471	Guo104	basidioma	Yiliang, YN	KT822242	KT822341	KT822148
*A*. *mellea*	/	HKAS 49004	Ge508	basidioma	Jiulong, SC	KT822245	KT822347	KT822150
*A*. *mellea*	/	HKAS 85599	Gt874	basidioma	Qujing, YN	KT822249	KT822342	KU378052
*A*. *mellea*	homothallic *A*. *mellea*	HKAS 86588	83003/2^2^	single spore	Japan	KT822246	KT822352	KT822151
*A*. *mellea*	(CBS G) homothallic *A*. *mellea*	HKAS 86590	00020/6^1^	strain	Yulong, YN	KT822251	KT822354	KT822158
*A*. *mellea*	(CBS G) homothallic *A*. *mellea*	HKAS 86591	01010/1^1^	strain	Shennongjia, HB	KT822252	KT822343	KT822156
*A*. *mellea*	(CBS G) homothallic *A*. *mellea*	HKAS 86592	02001/5^1^	strain	Kuankuoshui, GZ	KT822253	KT822344	KT822154
*A*. *mellea*	(CBS G) *homothallic A*. *mellea*	HKAS 86593	04054/14^1^	strain	Tianmushan, ZJ	KT822254	KT822345	KT822155
*A*. *mellea*	(CBS G) homothallic *A*. *mellea*	HKAS 86594	04055/9^1^	strain	Tianmushan, ZJ	KT822244	KT822349	KT822157
*A*. *mellea*	(CBS G) homothallic *A*. *mellea*	HKAS 86597	99044/2^1^	strain	Kunming, YN	KT822255	KT822351	KT822149
*A*. *mellea*	homothallic *A*. *mellea*	HKAS 86598	PFD84-103^2^	single spore	Africa	KT822248	KT822348	KT822159
*A*. *mellea*	homothallic *A*. *mellea*	HKAS 86599	PFD87-39^2^	single spore	Muguga, Kenya	KT822250	KT822350	KT822152
*A*. *mellea*	homothallic *A*. *mellea*	HKAS 86600	PFD87-41^2^	single spore	Kenya	KT822247	KT822355	KT822153
*A*. *mellea*	heterothallic *A*. *mellea*	HKAS 86611	B275^2^	tester strain	New Hampshire, US	KT822256	KT822353	KT822161
*A*. *mellea*	(CBS K) heterothallic *A*. *mellea*	HKAS 86612	00109/4^1^	single spore	Yulong, YN	KT822243	KT822346	KT822160
*A*. *nabsnona*	/	HKAS 85499	Guo316	basidioma	Yiliang, YN	KT822265	KT822410	KT822189
*A*. *nabsnona*	/	HKAS 85500	Guo317	basidioma	Yiliang, YN	KT822266	KT822413	KT822190
*A*. *nabsnona*	/	HKAS 86544	ZT-1^d^	commercial strain	Yiliang, YN	KT822270	KT822412	KT822191
*A*. *nabsnona*	/	HKAS 85523	Gt798	basidioma	Yiliang, YN	KT822333	KT822411	KT822192
*A*. *ostoyae*	(CBS D) *A*. *ostoyae*	HKAS 86579	96043/11^1^	single spore	Dunhua, JL	KT822310	KT822428	KT822215
*A*. *ostoyae*	(CBS D) *A*. *ostoyae*	HKAS 86580	96044/24^1^	single spore	Dunhua, JL	KT822311	KT822430	KT822216
*A*. *ostoyae*	(CBS D) *A*. *ostoyae*	HKAS 86581	97058B/16^1^	single spore	Dunhua, JL	KT822292	KT822429	KT822217
*A*. *ostoyae*	(CBS D) *A*. *ostoyae*	HKAS 86582	98+3/4^1^	single spore	Dunhua, JL	KT822307	KT822431	KT822177
*A*. *sinapina*	(CBS A) *A*. *sinapina*	HKAS 86566	96015/39^1^	single spore	Changbaishan, JL	KT822323	KT822422	KT822208
*A*. *sinapina*	(CBS A) *A*. *sinapina*	HKAS 86567	96037/12^1^	single spore	Changbaishan, JL	KT822272	KT822423	KT822211
*A*. *sinapina*	(CBS A) *A*. *sinapina*	HKAS 86568	99116/7^1^	single spore	Yichun, HLJ	KT822262	KT822424	KT822219
*A*. sp.	CBS N	HKAS 86620	02066/3^1^	single spore	Emeishan, SC	KT822317	KT822389	KT822236
*A*. sp.	CBS J	HKAS 86607	00112/5^1^	single spore	Yulong, YN	KT822330	KT822358	KT822210
*A*. *tabescens*	(CBS I) *A*. *tabescens*	HKAS 86603	99122/13^1^	single spore	Beijing	KT822240	KT822441	KT822339
*A*. *tabescens*	*A*. *tabescens*	HKAS 86604	CT1097.3^2^	tester strain	Messina, Italy	KT822338	KT822440	KT822239
*A*. *tabescens*	*A*. *tabescens*	HKAS 86605	90158^2^	tester strain	Slovenia	KT822337	KT822439	KT822238
Lineage 1	/	HKAS 85457	Guo90	basidioma	Yiliang, YN	KT822271	KT822385	KT822224
Lineage 1	/	HKAS 85519	Gt794	basidioma	Yiliang, YN	KT822335	KT822373	KT822225
Lineage 1	/	HKAS 85527	Gt802^c^	basidioma	Yiliang, YN	KT822322	KT822374	KT822227
Lineage 1	/	HKAS 85551	Gt826^c^	basidioma	Weixin, YN	KT822313	KT822382	KT822231
Lineage 1	/	HKAS 85575	Gt850^c^	basidioma	Weixin, YN	KT822314	KT822383	KT822230
Lineage 1	/	HKAS 85581	Gt856^c^	basidioma	Weixin, YN	KT822336	KT822392	KT822226
Lineage 1	CBS L	HKAS 86615	00126/2^1^	single spore	Kuankuoshui, GZ	KT822315	KT822384	KT822222
Lineage 1	CBS N	HKAS 86621	02069/5^1^	single spore	Emeishan, SC	KT822306	KT822386	KT822228
Lineage 1	CBS N	HKAS 86622	02071/2^1^	single spore	Emeishan, SC	KT822316	KT822390	KT822229
Lineage 2	/	HKAS 86551	Guo282^b^	rhizomorphs	Yiliang, YN	KT822279	KT822367	KT822183
Lineage 2	/	HKAS 86554	Guo322^b^	rhizomorphs	Yiliang, YN	KT822260	KT822366	KT822186
Lineage 2	/	HKAS 86556	Guo364^d^	commercial strain	Yiliang, YN	KT822259	KT822370	KT822187
Lineage 2	/	HKAS 86557	Guo365^d^	commercial strain	Yiliang, YN	KT822301	KT822368	KT822184
Lineage 2	/	HKAS 86543	SN4^d^	commercial strain	Yiliang, YN	KT822334	KT822369	KT822185
Lineage 2	CBS O	HKAS 86623	02072/23^1^	single spore	Emeishan, SC	KT822318	KT822363	KT822188
Lineage 3	/	HKAS 85449	Guo82	basidioma	Yiliang, YN	KT822299	KT822387	KT822176
Lineage 3	/	HKAS 86548	Guo278^b^	rhizomorphs	Yiliang, YN	KT822296	KT822395	KT822173
Lineage 3	/	HKAS 86552	Guo286^a^	rhizomorphs	Yiliang, YN	KT822297	KT822393	KT822220
Lineage 3	/	HKAS 86553	Guo287	basidioma	Yiliang, YN	KT822298	KT822394	KT822174
Lineage 3	CBS L	HKAS 86613	00125/1^1^	single spore	Kuankuoshui, GZ	KT822319	KT822388	KT822232
Lineage 3	CBS L	HKAS 86614	00125/2^1^	single spore	Kuankuoshui, GZ	KT822305	KT822391	KT822221
Lineage 3	/	HKAS 86555	Guo324^b^	rhizomorphs	Yiliang, YN	KT822302	KT822381	KT822175
Lineage 4	/	HKAS 51692	Yang4881	basidioma	Baiyu, SC	KT822269	KT822415	KT822163
Lineage 4	/	HKAS 51046	Ge1461	basidioma	Daocheng, SC	KT822257	KT822356	KT822162
Lineage 4	/	HKAS 85594	Gt869	basidioma	Yiliang, YN	KT822274	KT822377	KT822172
Lineage 4	/	HKAS 45820	Yang4434	basidioma	Changdu, Tibet	KT822258	KT822380	-
Lineage 4	CBS H	HKAS 86601	99012/1^1^	single spore	Yichun, HLJ	KT822280	KT822357	KT822166
Lineage 4	CBS H	HKAS 86602	00019/4^1^	single spore	Yulong, YN	KT822308	KT822378	KT822167
Lineage 4	CBS J	HKAS 86606	00107/2^1^	single spore	Yulong, YN	KT822281	KT822359	KT822171
Lineage 4	CBS J	HKAS 86608	00115/3^1^	single spore	Xianggelila, YN	KT822282	KT822375	KT822165
Lineage 4	CBS J	HKAS 86609	00117/7^1^	single spore	Xianggelila, YN	KT822331	KT822372	KT822169
Lineage 4	CBS J	HKAS 86610	02049/2^1^	single spore	Xinyuan, XJ	KT822332	KT822371	KT822164
Lineage 4	(CBS M) *A*. *borealis*	HKAS 86618	01018/10^1^	single spore	Shennongjia, HB	KT822321	KT822376	KT822170
Lineage 4	(CBS M) *A*. *borealis*	HKAS 86619	01019/1^1^	single spore	Shennongjia, HB	KT822295	KT822362	KT822168
Lineage 5	CBS C	HKAS 86574	93022/13^1^	single spore	Changbaishan, JL	KT822324	KT822361	KT822200
Lineage 5	CBS C	HKAS 86575	96018/7^1^	single spore	Changbaishan, JL	KT822327	KT822364	KT822201
Lineage 5	CBS C	HKAS 86576	99109/6^1^	single spore	Yichun, HLJ	KT822326	KT822360	KT822234
Lineage 5	CBS C	HKAS 86577	99110/6^1^	single spore	Yichun, HLJ	KT822267	KT822379	KT822235
Lineage 5	CBS C	HKAS 86578	99115/10^1^	single spore	Yichun, HLJ	KT822325	KT822365	KT822209
Lineage 6	/	HKAS 86558	A9^d^	commercial strain	Korea	KT822285	KT822407	KT822198
Lineage 6	/	HKAS 86559	Guo343^d^	commercial strain	Hanzhong, SX	KT822289	KT822406	KT822197
Lineage 6	/	HKAS 86560	Jing-234^d^	commercial strain	SX	KT822264	KT822408	KT822212
Lineage 6	/	HKAS 86564	Guo341^d^	commercial strain	HB	KT822284	KT822405	KT822196
Lineage 6	/	HKAS 85567	Gt842^c^	basidioma	Weixin, YN	KT822268	KT822399	KT822213
Lineage 6	/	HKAS 85572	Gt847^c^	basidioma	Weixin, YN	KT822273	KT822400	KT822207
Lineage 6	(CBS B) *A*. *gallica*	HKAS 86571	02147/1^1^	single spore	Mudanjiang, HLJ	KT822288	KT822404	KT822205
Lineage 6	(CBS B) *A*. *gallica*	HKAS 86573	96011/25^1^	single spore	Changbaishan, JL	KT822300	KT822398	KT822202
Lineage 6	/	HKAS 86561	K19^d^	commercial strain	China	KT822329	KT822401	-
Lineage 6	/	HKAS 86563	Guo342^d^	commercial strain	HB	KT822286	KT822397	KT822199
Lineage 6	/	HKAS 45821	Yang4445	basidioma	Kunming, YN	KT822261	KT822396	KT822194
Lineage 6	(CBS B) *A*. *gallica*	HKAS 86570	01106/7^1^	single spore	Beian, HLJ	KT822320	KT822402	KT822204
Lineage 6	(CBS B) *A*. *gallica*	HKAS 86572	96011/16^1^	single spore	Changbaishan, JL	KT822328	KT822403	KT822206
Lineage 7	/	HKAS 83303	Qin886	basidioma	Longling, YN	KU378047	KT822437	KU378049
Lineage 7	/	HKAS 83361	Qin944	basidioma	Longling, YN	KU378048	KT822436	KU378050
Lineage 7	/	HKAS 86541	Xhwang3394	basidioma	Lincang, YN	KT822304	KT822435	KT822237
Nag. E	/	HKAS 86549	Guo279^b^	rhizomorphs	Yiliang, YN	KT822275	KT822433	KT822195
Nag. E	/	HKAS 86550	Guo281^b^	rhizomorphs	Yiliang, YN	KT822276	KT822434	KT822233
North American *A*. *cepistipes*	CBS F	HKAS 86586	97033/1^1^	single spore	Changbaishan, JL	KT822263	KT822416	KT822223

^1^ the isolates were previously assigned to biological species by Qin et al. [11].

^2^ tester strains.

The samples associated with Tianma

^a^ rhizomorphs attached to the tuber surfaces

^b^ rhizomorphs collected from inoculum (wood infected with *Armillaria* strains by farmers) at the Tianma plantations

^c^ basidiomata collected from inoculum (wood infected with *Armillaria* strains by farmers) at the Tianma plantations

^d^ commercial strains of *Armillaria* used for the cultivation of Tianma

Abbreviations: CBS = Chinese biological species; HKAS = the Herbarium of Cryptogams of the Kunming Institute of Botany (KIB); Chinese Academy of Sciences; SC = Sichuan Province; YN = Yunnan Province; HB = Hubei Province; JL = Jilin Province; HLJ = Heilongjiang Province; LN = Liaoning Province; GZ = Guizhou Province; ZJ = Zhejiang Province; XJ = Xinjiang Province; SX = Shaanxi Province

### DNA isolation, PCR, DNA sequencing and alignments

DNA was directly extracted from dry specimens or cultures using the cetyltrimethylammonium bromide (CTAB) method [38]. Primers ITS1F and ITS4B [39] were used to amplify the 5.8S rRNA gene and the flanking ITS regions, and primer pairs EF595F/EF1160R [40] or 983F/1567R [41] were used to amplify *tef1-α*. For *β*-*tubulin*, primer pairs TubF (5′- GGTGCGGGTAACTGGGC -3′) and TubR (5′- GAGGCAGCCATCATGTTCTT -3′) [42] were used in the amplifications. PCR was performed in a total volume of 25 μl containing 2.5 μl of PCR reaction buffer, 0.5 μl of dNTP mixture (0.2 mM), 1 μl of each primer (5 μM), 1 U of Taq polymerase, and 1 μl of DNA template. PCR reactions were conducted on an ABI 2720 Thermal Cycler (Applied Biosystems, Foster City, CA, USA) under the following reaction conditions: predenaturation at 94°C for 3 min, followed by 35 cycles of denaturation at 94°C for 40 s, annealing at 50°C (for ITS), 52°C (for *tef1*-*α*) and 53°C (for *β*-*tubulin*) for 40 s, and elongation at 72°C for 90 s. A final elongation at 72°C for 6 min was included after the cycles. We run 3–5 μl of the PCR products on a 2% agarose gel to verify correct amplification. PCR products were purified with a Gel Extraction & PCR Purification Combo Kit (Spin-column) (Bioteke, Beijing, China). The purified products were then sequenced in both directions on an ABI-3730-XL DNA Analyzer (Applied Biosystems, Foster City, CA, USA) using the same primers as in the PCR amplifications. For most of the samples, PCR amplification of ITS and *β*-*tubulin* yielded a single strong band. Amplicons ranged in size between ~1,000 bp for ITS and ~900 bp for *β*-*tubulin*. Amplification of the *tef1*-*α* region yielded a strong band of about 600 bp for all of the samples. PCR products that could not be sequenced successfully were cloned into a PMD18-T vector (Takara, Japan) and then sequenced with primers M13F (5′ -GTAAAACGACGGCCAGTGAA-3′) and M13R (5′ -CAGGAAACAGCTATGACCAT-3′). The contiguous sequences were assembled with SeqMan implemented in Lasergene v7.1 (DNASTAR Inc., USA).

Six matrices were compiled for different purposes. The first four matrices, namely, ITS, *tef1*-*α* (EF1-I matrix), *β*-*tubulin* and the three-locus matrices, consisted of sequences generated in this study. They were used to delimit phylogenetic lineages of *Armillaria* in China. The four matrices contained sequences obtained from nine tester strains, which have been used by other authors to perform mating tests with Chinese isolates to identify biological species [11, 12, 43]. We used these sequences to achieve broader sampling for establishing the relationships between the biological species and phylogenetic lineages. A fifth matrix, named here as the comprehensive *tef1-α* matrix (EF1-II matrix), comprised *tef1-α* sequences generated in this study (from 101 collections) and 81 sequences retrieved from the GenBank (NCBI; http://blast.ncbi.nlm.nih.gov/; S1 Table). These sequences were selected based on two criteria: i) they were representative of species isolates from the Northern Hemisphere (Europe and North America) and East Africa, and ii) mating tests had been performed on these isolates. This 182-collection dataset was used to identify the relationships among all of our samples and known related samples in the GenBank.

We found that some diploid individuals contained many heterozygous sites (more than five sites) in the *tef1*-*α* region based on the alignment of EF1-II. In these cases, the program PHASE 2.0 (http://www.bioinf.manchester.ac.uk/resources/phase/) was used to resolve the haplotype of these heterozygous samples using the default parameter settings [44]. Therefore, a sixth matrix (EF1-III matrix), which was based on the EF1-II matrix and contained these haplotypes, was generated to illuminate the phylogenetic relationships among them. For all of the matrices, *A*. *ectypa* (Fr.) Lamoure was selected as the outgroup based on a previous study [22].

Each of the ITS, *β*-*tubulin* and EF1-I, II, and III matrices were aligned in MAFFT v6.853 with the auto strategy [45], and manually optimized in BioEdit v7.0.9 [46]. The three-locus matrix was compiled by concatenating the sequences of the ITS, EF1-I, and *β*-*tubulin* matrices in Phyutility 2.2 [47]. The regions containing insertions and deletions (indels) were included in the analysis, with gaps treated as missing data.

To test whether potentially ambiguous sites in the original alignments would add noise to the phylogenetic analyses, we first used the on-line Gblocks v0.91b server (http://molevol.cmima.csic.es/castresana/Gblocks_server.html) to select sites by specifying less stringent conditions, i.e., allowing smaller final blocks, gap positions within the final blocks and less strict flanking positions. The generated alignment of ITS, *tef1-α* and *β*-*tubulin* retained 93%, 99% and 96% original characters, respectively. The excluded sites in the *tef1-α* and *β*-*tubulin* alignments were all within introns. However, the introns could be unambiguously aligned and had many parsimony-informative characters. In addition, comparison of the trees generated from the processed and original alignments showed that the original alignments produced even better topologies with higher support values. Many authors consider that there is a substantial loss of information upon the removal of any fragments from sequences that have already been obtained [48, 49]. Therefore, in our final analyses, we included all of the characters. In the three-locus matrix, the unavailable *β-tubulin* sequences of three samples were treated as missing data in the phylogenetic analyses. The alignments of the six matrices are available from TreeBASE under accession no. 18700.

### Phylogenetic analyses

The best-fitted substitution model for each matrix was determined through MrModeltest v2.3 [50] based on the Akaike information criterion (AIC). GTR+I+G, SYM+I+G and HKY+I+G were selected as the best models for the three-locus and ITS matrices, the three *tef1*-*α* matrices (EF1-I, II and III), and the *β*-*tubulin* matrix, respectively. Maximum likelihood (ML) analyses, Bayesian inference (BI) and maximum parsimony (MP) bootstrap analyses were conducted for the three-locus, EF1-II and EF1-III matrices. For the phylogenetic analyses based on the ITS, *tef1*-*α* (EF1-I) and *β*-*tubulin* matrices, only ML and BI were used. ML analyses were carried out using the program RaxML 7.0.4 [51] as implemented in the Cyberinfrastructure for Phylogenetic Research (CIPRES) Web Portal 1.0 (http://www.phylo.org/sub). The support values for each node were assessed using a nonparametric bootstrapping with 1,000 replicates. BI was conducted in MrBayes 3.1.2 [52] with two independent runs of one cold and three heated chains. Runs were performed for 50 million generations with trees sampled every 100 generations. Chain convergence was determined using Tracer v1.5 (http://tree.bio.ed.ac.uk/software/tracer/) to confirm sufficiently large ESS values (> 200). The sampled trees were subsequently summarized after omitting the first 25% of trees as burn-in using the “sump” and “sumt” commands implemented in MrBayes. The default parameters were used for both methods, with convergence and burn-in assessed according to the authors’ recommendations [51, 52]. The three-locus matrix was divided into three partitions, and we conducted partitioned mixed-model analysis that allowed us to estimate the model parameters separately in both BI and ML analyses. MP analyses were performed in PAUP [53] based on a heuristic search of 1,000 replicates with random stepwise addition using tree-bisection-reconnection branch swapping and starting with trees obtained by the stepwise addition of sequences. All of the characteristics were equally weighted and gaps were treated as missing data. Parsimony bootstrap analyses [54] with 1,000 replicates were subsequently performed using the fast bootstrap option to evaluate the robustness of the MP trees.

### Phylogenetic lineage recognition

Phylogenetic lineages of *Armillaria* from China were delimited based on two criteria proposed by Dettman et al. [18, 19]. A clade was assigned to a phylogenetic lineage if it fulfilled either of the following two criteria: (i) genealogical concordance: the clade was present in the majority (2/3) of the single-locus genealogies; or (ii) genealogical non-discordance: the clade was well supported in at least one single-locus genealogy (for ML, bootstrap proportions (BP) ≥ 70%, Bayesian posterior probability (PP) ≥ 0.95), and it was not contradicted in any other single-locus genealogy at the same level of support. A conflict was assumed to be significant when two different relationships (one monophyletic and the other non-monophyletic) for the same set of taxa were supported either with ML-BP ≥ 70% or BI-PP ≥ 0.95. For any clade which failed to obtain multigene sequences from at least two samples, it was also accepted as a potential independent phylogenetic lineage if it showed long branches as sequence variation from its sister groups.

## Results

### Phylogenetic analyses

**Fig 1:**
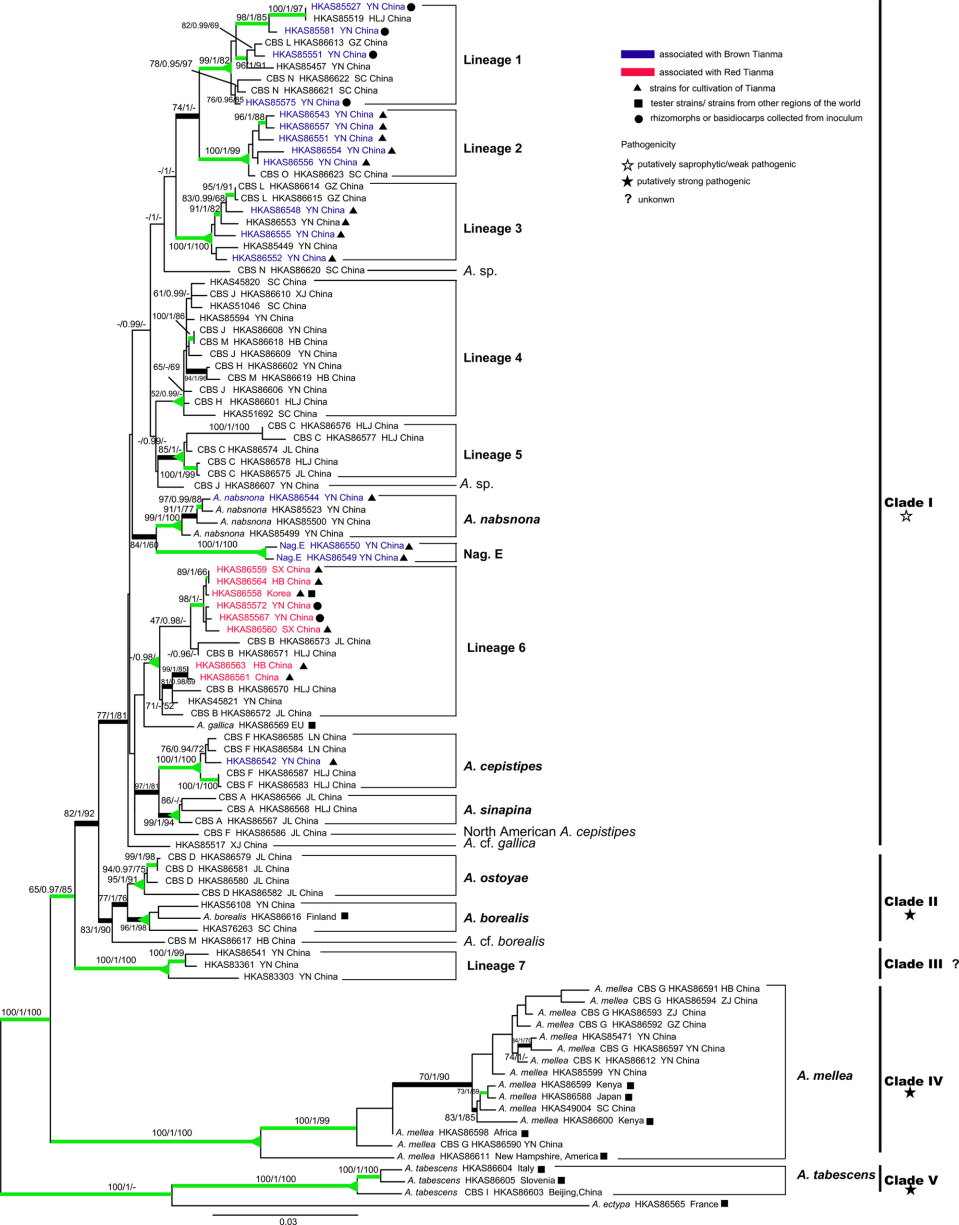
Phylogenetic tree generated from the three-locus (ITS, *tef1-α* and *β-tubulin*) dataset. The labels on the right of the phylogram indicate groups identified by phylogenetic analyses. The values of the bootstrap frequencies of ML (BP ≥ 70%), posterior probability (PP ≥ 0.95) and maximum parsimony bootstraps (PB ≥ 70%) are shown above the nodes. Thick black branches indicate high support in the analyses (ML-BP ≥ 70%, BI-PP ≥ 0.95). Green branches were concordantly supported by the majority of the loci (genealogical concordance), or were well supported by at least one locus and not contradicted by any other locus (genealogical non-concordance). Triangles at nodes indicate that all taxa united by it belong to the same phylogenetic lineage (see text for details). Taxon labels indicate strain number and geographic source. If a strain was originally identified as a CBS (Chinese biological species), the CBS taxon is listed before the strain number.

**Fig 2:**
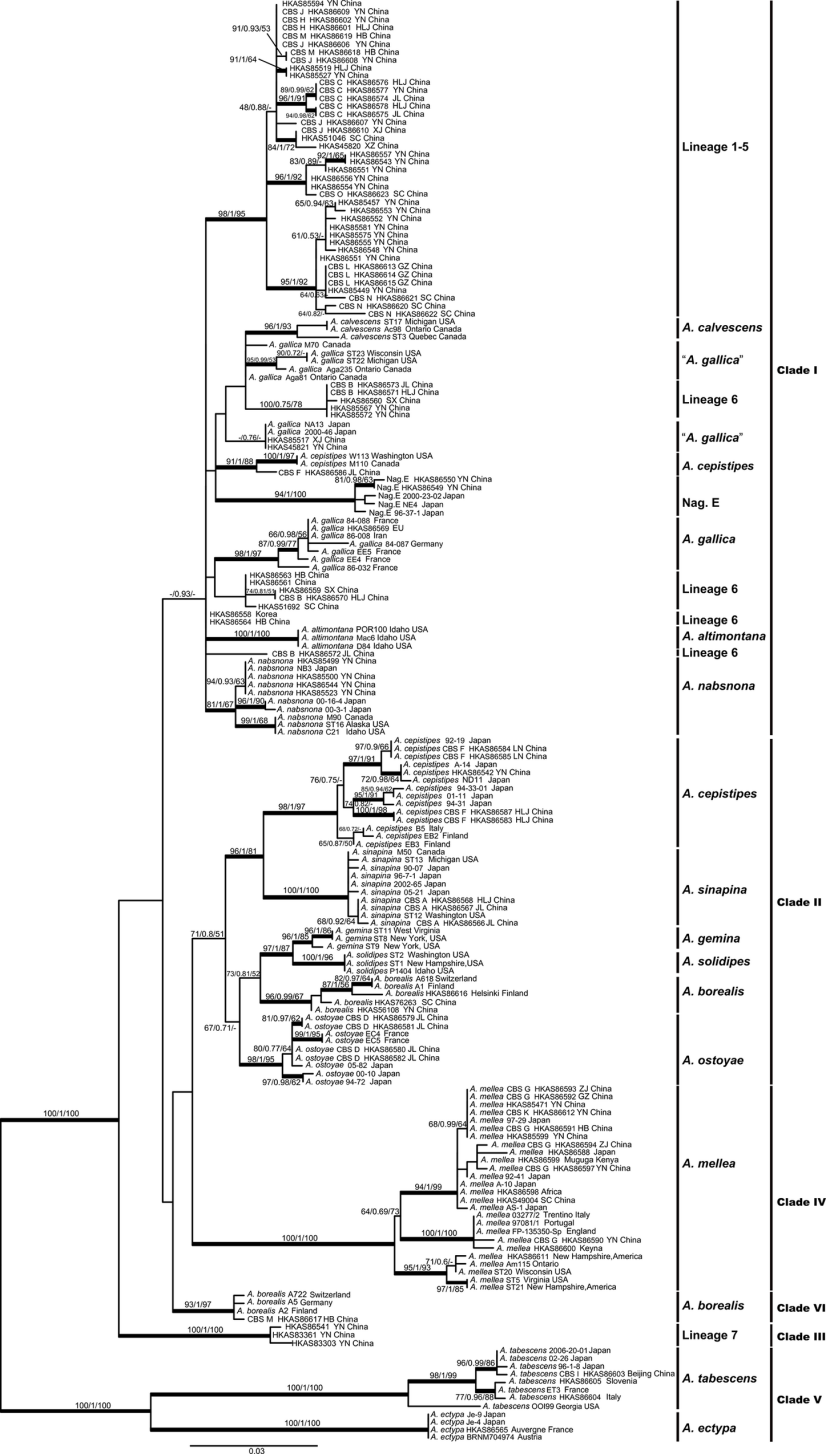
Phylogenetic relationships between samples of *Armillaria* inferred from *tef1-α* sequences using ML analysis. Branch support values are indicated by numbers above branches (ML-BP/BI-PP/MP-PB). Thick black branches received high support in the analyses (ML-BP ≥ 70%, BI-PP≥ 0.95). The accession numbers for the sequences retrieved from the GenBank database are listed in S1 Table.

The ITS, EF1-I, and *β*-*tubulin* matrices included 101, 101 and 98 sequences, respectively. The number of nucleotides/characters (and the percentages of parsimony-informative characters) in these three matrices were 910 (12.6%), 515 (28.7%) and 850 (20.7%), respectively. For each of these three matrices, ML and BI analyses yielded similar topologies. The phylogenetic trees generated from ML analyses are shown in S1 Fig. Overall, branch resolution and support in the ITS phylogeny were lower, and only a few branches were supported by both the bootstrap and posterior probabilities, while better branch resolution and support were obtained for the trees generated from the EF1-I and *β*-*tubulin* sequences. Several well supported lineages appeared in common between the two single-locus genealogies, i.e., *A*. *mellea*, *A*. *cepistipes*, putative new Lineages 2 and 7, *A*. *tabescens* and Nag. E (S1 Fig). Concurrently, the *β*-*tubulin* data showed supported conflicts with *tef1-α* with regard to the placement of several phylogenetic lineages, i.e., *A*. *ostoyae*, *A*. *sinapina* and *A*. *borealis* (S1 Fig). Nevertheless, the phylogenetic signal in the individual ITS and *tef1*-*α* datasets was so strong that the incongruence from *β*-*tubulin* was eliminated in the three-locus analyses. The ML trees of the three-locus and EF1-II matrices are presented in Figs 1 and 2, respectively. In Fig 2, we label the clade including samples HKAS86579, HKAS86581, HKAS86580 and HKAS86582 as *A*. *ostoyae*, because our samples were nested in the same clade as the GenBank-retrieved sequences of European *A*. *ostoyae*, and this name is more popularly acknowledged than its putative synonym *A*. *solidipes* (Fig 2). Phylogenetic analyses based on the three-locus dataset revealed five major clades (Fig 1), while those based on the EF1-II matrix indicated six major clades (Fig 2). The major differences between the trees inferred from the two matrices were as follows: (i) *A*. cf. *borealis*, which clustered into Clade II in the tree generated from the three-locus dataset (Fig 1), formed a single clade (VI) in the phylogenetic tree from the EF1-II matrix; and (ii) *A*. *cepistipes* and *A*. *sinapina*, which clustered into Clade I (Fig 1) in the tree of the three-locus matrix, were placed in Clade II in the EF1-II phylogeny (Fig 2).

**Fig 3:**
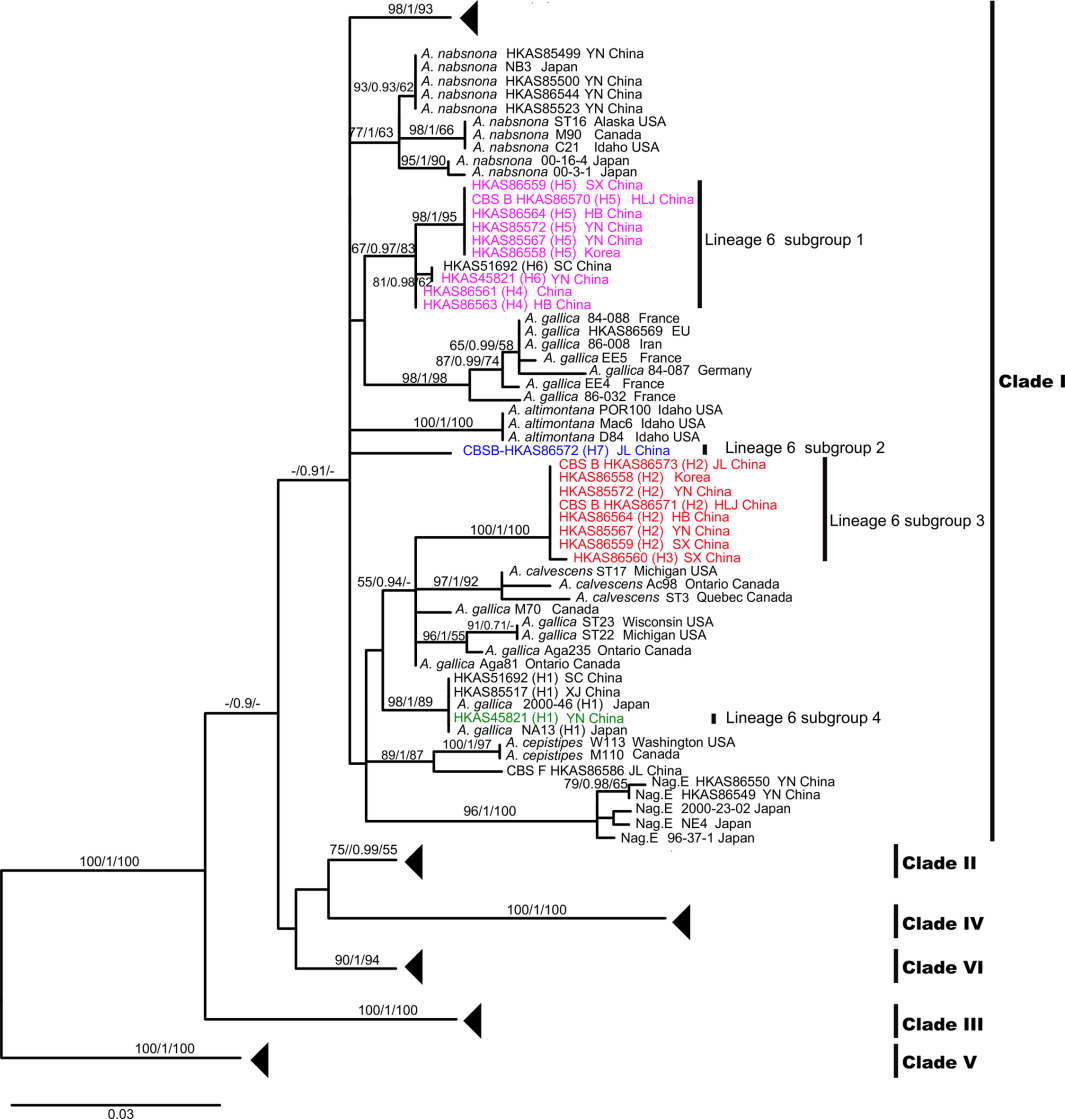
Phylogeny of *Armillaria* samples inferred from *tef1-α* DNA sequences using ML analysis to show the intraspecific level within Lineage 6. Branch support values are indicated by numbers above branches (ML-BP/BI-PP/MP-PB). The nodes appearing in common between Fig 3 and Fig 2 are indicated by triangles. The species names labeled on our collections correspond to Fig 1. Seven samples with heterozygous sites (HKAS45821, HKAS51692, HKAS86558, HKAS86559, HKAS86564, HKAS85567 and HKAS85572) were analyzed using PHASE 2.0 and every isolate was divided into two haploid genotypes. The four subgroups of Lineage 6 were highlighted in different colors.

The PHASE analyses inferred four haplotypes within the seven heterozygous strains (HKAS51692, HKAS45821, HKAS86559, HKAS86564, HKAS85572, HKAS85567 and HKAS86558) (S2 Fig). The phylogeny containing these haplotypes instead of heterozygous sequences is shown in Fig 3. The phylogeny (EF1-III) displayed an identical topology to the EF1-II phylogeny except for the shallow nodes surrounded by the five heterozygous samples: therefore, the deep nodes appearing in common between EF1-III and EF1-II tree are indicated by triangles.

### Phylogenetic lineage recognition

At least 15 phylogenetic lineages of *Armillaria* were recognized from China. Among them, four (*A*. *mellea*, *A*. *tabescens*, Nag. E and Lineage 7) were strongly supported by all three genealogies (ITS, EF1-I and *β-tubulin*), two (*A*. *cepistipes* and Lineage 2) were strongly supported as monophyletic by ML-BP (≥ 70%) and Bayesian-PP (≥ 0.95) in two of the individual gene trees (S1 Fig; Table 2), and four lineages (*A*. *nabsnona*, Lineages 1, 3 and 4) were resolved as monophyletic in one of the three individual gene trees and were not rejected by the other two matrices (Table 2). The five phylogenetic lineages (Lineage 5, Lineage 6, *A*. *sinapina*, *A*. *ostoyae* and *A*. *borealis*) only formed monophyletic groups in the *tef1-α* gene tree. Except HKAS86573, 11 samples of Lineage 6 formed a monophyletic group (93/1.00) in the *β-tubulin* tree (S1 Fig). The sequences of this lineage lacked significant genetic differentiation, and the genealogical patterns were common among the three loci, so it is rational to collapse these into a single phylogenetic lineage. The branches of HKAS86569 (European *A*. *gallica*) were relatively long in all three single-locus genealogies and the combined tree revealed that Lineage 6 and European *A*. *gallica* were probably different phylogenetic lineages, accordingly (S1 Fig; Fig 1). The other five phylogenetic lineages were represented by single collections (Fig 1), thus their monophyly could not be tested. However, the five singletons (HKAS86620, HKAS86607, HKAS 85517, HKAS 86586 and HKAS 86617) showed relatively long branches compared with their sister groups: therefore, they were considered to be genetically divergent from their sisters (Fig 1, Clades I and II). Among the 15 phylogenetic lineages as determined by the corcordance and/or non-disconcordance of the three genealogies, the monophyly of seven lineages is highly supported in the *tef1-α* genealogy and monophyly of 10 lineages is highly supported in the *β-tubulin* genealogy (S1 Fig; Fig 1; Table 2).

**Table 2:** Support values for *Armillaria* lineages recognized by genealogical concordance in analyses of individual gene partitions and in the combined three-locus dataset. Values are shown as ML-BP/Bayesian-PP. Missing values (-) apply to non-monophyly.

Independent evolutionary lineage^1^	ITS	*tef1-α*	*β-tubulin*	Criteria satisfied	Combined three-locus data ^2^
*A*. *borealis*	-/-	98/1.00	-/-	n	96/1.00/98
*A*. *cepistipes*	-/-	99/1.00	96/1.00	①	100/1.00/100
*A*. *mellea*	100/1.00	100/1.00	100/1.00	①②	100/1.00/100
*A*. *nabsnona*	-/-	100/1.00	-/-	②	99/1.00/100
*A*. *ostoyae*	-/-	99/1.00	-/-	n	95/1.00/91
*A*. *sinapina*	-/-	100/1.00	-/-	n	99/1.00/94
Lineage 1	-/-	-/-	81/0.97	②	99/1.00/82
Lineage 2	-/-	95/1.00	100/1.00	①	100/1.00/99
Lineage 3	-/-	-/-	100/1.00	②	100/1.00/100
Lineage 4	-/-	-/-	100/1.00	②	52/0.99/-
Lineage 5	99/1.00	91/1.00	-/-	n	85/1.00/-
Lineage 6	-/-	-/-	93/1	n	41/0.98/-
Lineage 7	93/1.00	100/1.00	100/1.00	①②	100/1.00/100
*A*. *tabescens*	98/1.00	100/1.00	100/1.00	①②	100/1.00/100
Nag. E	100/1.00	99/1.00	96/1.00	①②	100/1.00/100

^1^ Support values not applicable for the following five potential lineages represented by single collections, which are therefore not included in the table: *A*. sp. (HKAS86620), *A*. sp. (HKAS86607), *A*. cf. *gallica* (HKAS85517), North American *A*. *cepistipes* (HKAS86586), *A*. cf. *borealis* (HKAS86617).

^2^ The maximum parsimony (MP) bootstrap analysis was added in the three-locus phylogeny.

① genealogical concordance, ② genealogical non-discordance.

“n” represents neither ① nor ② was fulfilled, but accepted as phylogenetic lineage, see text for details.

### Phylogenetic lineages of *Armillaria* associated with Tianma

A total of 23 isolates associated with Tianma were obtained from our study. The collections associated with Tianma belonged to seven independent phylogenetic lineages, including Lineages 1 to 3, *A*. *nabsnona*, Nag. E, *A*. *cepistipes*, and Lineage 6 (Fig 1). Nag. E was recorded to be associated with Tianma for the first time by this study. Among these seven phylogenetic lineages, the strains of Lineage 2 and Lineage 6 are the most commonly used in the cultivation of Tianma (authors’ personal observations), accounting for 22% (5 out of 23) and 30% (7 out of 23) of the strains, respectively (Fig 1).

## Discussion

### Phylogenetic lineage diversity of *Armillaria* in China and the assignment of 14 CBS

Our study revealed a high diversity of *Armillaria* species in China. At least 15 phylogenetic lineages were recognized (Fig 1), 10 of which were represented by two or more collections and could be supported by the GCPSR criteria of Dettman et al. [19]. The grouping of the samples of the remaining five lineages did not strictly satisfy either genealogical concordance or genealogical non-disconcordance. The major conflict was between the genealogies of *tef1*-*α* and *β-tubulin*. For example, the samples of Lineage 5 were well supported in the *tef1*-*α* tree but collapsed into two subgroups in the *β-tubulin* genealogy (S1 Fig). The monophyly of four isolates of *A*. *ostoyae* was supported in the *tef1*-*α* tree. However, these isolates collapsed into two subgroups in the *β-tubulin* tree, one group was nested with a subgroup of Lineage 5 and the other one was cluster with sample of *A*. *borealis*, respectively. The samples of *A*. *sinapina* formed a monophyletic clade in the *tef1*-*α* genealogy but split into three divergent subgroups in the *β-tubulin* tree. Nevertheless, considering the high support values in the combined tree, we took these clades as independent phylogenetic lineages. The remaining five potential phylogenetic lineages were each represented by a single strain (HKAS86620, HKAS86607, HKAS85517, HKAS86586 and HKAS86617) but showed relatively long branches, seeming to suggest obvious genetic divergence from their close relatives (Fig 1).

**Fig 4:**
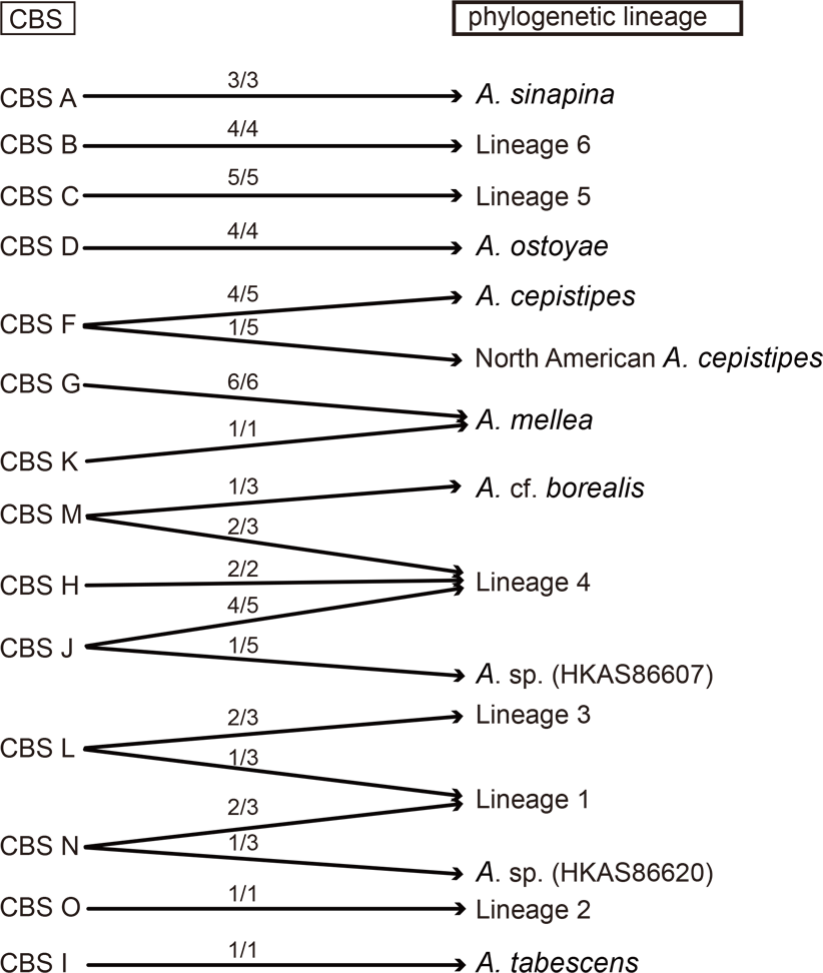
The link between 14 CBS (Chinese Biological Species) and phylogenetic lineages identified in this study. CBS were recognized in previous studies and phylogenetic lineages were delimited in this study. The m/n on the arrows between “CBS” and “phylogenetic lineage” means that m of n isolates of a CBS were assigned to phylogenetic lineages.

The 14 CBS proposed based on mating tests could be assigned to specific phylogenetic lineages with the help of our multigene data. Coetzee et al. [30] analyzed these CBS using the sequences of *tef1*-*α* and IGS-1. They revealed four main phylogenetic groups and named them the “*A*. *ostoyae*”, “*A*. *gallica*”, “*A*. *tabescens*” and “*A*. *mellea*” clusters. However, no clear assignment of these CBS was provided in their study. In our analyses, some unexpected relationships between CBS and phylogenetic lineages were indicated. Two types of correspondence among the CBS and the phylogenetic lineages were suggested by our data (Fig 4). The first type showed clear relationships among the phylogenetic lineages and the CBS, and could be divided into three subtypes: (i) one CBS to one phylogenetic lineage; this included CBS A, B, C, D, O and I, which could be assigned to *A*. *sinapina*, Lineage 6, Lineage 5, *A*. *ostoyae*, Lineage 2 and *A*. *tabescens*, respectively; (ii) one CBS to two phylogenetic lineages; this was represented by CBS F, which was divided into two subgroups, provisionally named *A*. *cepistipes* and North American *A*. *cepistipes*; and (iii) two CBS to one phylogenetic lineage; this included CBS G and K, which were merged into *A*. *mellea*. The second type was much more complicated. This type included two groups of CBS: (i) the group consisting of CBS M and J, both of which were separated into two phylogenetic lineages, but shared Lineage 4, represented by CBS H; and (ii) the group consisting of CBS L and N, both of which were divided into two lineages, but shared Lineage 1.

Our multigene data provided several notable new insights into the treatment of the CBS. Some incompatible CBS presented the same phylogenetic lineage. For example, the homothallic strains of CBS G have been reported as almost incompatible with the heterothallic strains of CBS K. However, they nested within the *A*. *mellea* clade with strong support (100/1/99) (Fig 1, Clade IV) in our phylogenetic analyses. Similar observations have also been made for other fungal groups. For example, the homothallic and heterothallic biotypes of the pathogen *Moniliophthora perniciosa* were not inter-fertile, presumably because genetic barriers have evolved since the biotypes diverged [55]. Thus, it is quite reasonable to accept CBS G and K as a single phylogenetic lineage. In contrast, some compatible strains of a biological species could be further divided into two or more phylogenetic lineages. For instance, CBS M, which has been demonstrated to be compatible with European *A*. *borealis*, could be divided into two monophyletic clades. The scenario whereby one biological species actually encompasses more than one phylogenetic lineage has been discussed in the context of European *Armillaria* collections [24]. A similar phenomenon was observed for CBS F. This biological species has been suggested to be conspecific to *A*. *singula*, which was originally described from Japan, on the basis of its basidioma morphology [11], or to represent a novel taxon closely related to *A*. *cepistipes*, *A*. *calvescens*, *A*. *gallica* and *A*. *sinapina*, based on phylogenetic analyses of the IGS-1 region [26]. Although no compatibility reaction emerged between the isolates of CBS F and European or North American *A*. *cepistipes* [32], most of the CBS F isolates were grouped with those of *A*. *cepistipes* from Japan and Europe into a common sub-clade with high support in the *tef1*-*α* tree, and the isolate HKAS86586 was unexpectedly grouped with the North American *A*. *cepistipes* (Fig 2). Thus, it is reasonable to identify the isolates of CBS F as *A*. *cepistipes* and the closely related “North American *A*. *cepistipes*”.

### Relationships between *Armillaria* from China and other regions of the world

By comparing *tef1*-*α* sequences from our study with sequences from the GenBank database, we explored the phylogenetic relationships between Chinese *Armillaria* isolates and those from other parts of the world. Of the recognized taxa, *A*. *mellea* and *A*. *tabescens* have been reported to be widely distributed in the Northern Hemisphere. However, we found significant genetic divergence among isolates of each of these two species from different continents (Figs 1 and 2), indicating genetic isolation among populations from different continents. Nevertheless, it is surprising that the homothallic *A*. *mellea* from Southwest China (HKAS86590) and East Africa (HKAS86600) were phylogenetically very close to those gathered from Europe (Fig 2). Furthermore, the African samples of homothallic *A*. *mellea* (HKAS86598 and HKAS86599) were grouped within a sub-clade of predominantly East Asian samples (Figs 1 and 2). This is similar to observations reported previously [15, 56]. The disjunction of closely related species of *Armillaria* between China and Africa is probably due to recent human-mediated introductions, which was also suggested by biogeographic studies of other fungi, such as *Favolaschia*, *Serpula* and *Pleurotus* [57–59]. *Armillaria gallica* has also been recorded from different continents of the Northern Hemisphere. Our study indicated that the samples recognized as *A*. *gallica* from East Asia and North America were genetically strongly divergent from European *A*. *gallica*. In total, two phylogenetic lineages could be plausibly inferred for “*A*. *gallica*” (Fig 2). Most of the samples from China previously considered as *A*. *gallica*, now represented a new phylogenetic lineage (Lineage 6) according to our analyses, and one specimen (HKAS85517) from Xinjiang (XJ) together with two *A*. *gallica* samples from Japan formed another clade, while the *A*. *gallica* from North America formed a separate clade (Fig 2). This is similar to previous reports [60]. In addition, significant heterozygous sites were detected in the *tef1*-*α* sequences of six strains within Lineage 6 (HKAS45821, HKAS86559, HKAS86564, HKAS85572, HKAS85567 and HKAS86558). After including these haplotypes in the phylogenetic analyses, we found that the two alleles of some strains (HKAS51692 and HKAS45821) were clustered into different phylogenetic lineages (Fig 3), indicating that hybridization could occur among different phylogenetic subgroups. Hybridization events commonly occurr in pathogenic fungi [61–64]. Interspecific hybridization has only been reported for three Basidiomycota: *Coniophora puteana* [65], *Flammulina velutipes* [66] and *Tricholoma scalpturatum* [67]. Even if direct evidence is still lacking, some *Armillaria* species may have interspecific hybridization potential because they already have a broad range of hosts.

The phylogenetic lineages shared between East Asia and Europe included *A*. *borealis*, *A*. cf. *borealis*, *A*. *ostoyae* and *A*. *cepistipes*. We found that the Chinese isolates of “*A*. *borealis*” encompassed two lineages (Figs 1 and 2). Similarly, European “*A*. *borealis*” has also been reported as two divergent lineages [60]. The isolates HKAS56108 and HKAS76263 were grouped into the *A*. *borealis* lineage, and the isolate HKAS86617 was grouped into *A*. cf. *borealis* (Fig 1). Samples of *A*. *ostoyae* from northeastern China, Japan and Europe were grouped into a highly supported lineage. Based on morphological observations, Burdsall & Volk [3] have proposed that *A*. *solidipes* Peck, originally described from North America, is an older name for *A*. *ostoyae*, originally described from Europe. Our phylogenetic analysis indicated that the North American *A*. *solidipes* was sister to its sympatric *A*. *gemina* rather than the Eurasian *A*. *ostoyae*. We thus provisionally assign the North American isolates to *A*. *solidipes* and the Eurasian isolates to *A*. *ostoyae*.

*Armillaria nabsnona*, North American *A*. *cepistipes* and *A*. *sinapina* are common in East Asia and North America. Isolates from coastal western North America, Japan and southwestern China exhibited great genetic differentiation within *A*. *nabsnona* and formed three subclades. The isolates from North America, Japan and northeastern China showed limited genetic variation in *A*. *sinapina*. The North American *A*. *cepistipes* is distributed in North America and Northeast China, and is genetically divergent from the East Asian–European *A*. *cepistipes* lineage (Fig 2). The relationships between “*A*. *cepistipes*” in North America and East Asia can only be elucidated when more collections are available.

The phylogenetic lineages restricted to East Asia were Nag. E and Lineage 6. Nag. E is a biological species previously identified in Japan by Ota et al. [68]. Phylogenetically, it is closely related to *A*. *nabsnona* (Clade I in Fig 2). Four isolates within Lineage 6 belong to CBS B, and CBS B was assigned to *A*. *gallica* previously. Prior to this study, *A*. *gallica* had also been recorded from China and Japan [11, 68, 69]. Interestingly, we found that the “*A*. *gallica*” from East Asia was genetically different from European *A*. *gallica*. In our phylogenetic analyses, the tester strain of *A*. *gallica* from Europe (HKAS86569) was genetically differentiated from samples of “*A*. *gallica*” from other regions (Figs 1 and 2). HKAS86569 formed a congruent genetic pattern (displayed as substitutions) in the alignments of three loci within the lineage, suggesting that it may represent a different phylogenetic species from Lineage 6 (Figs 1 and 2; S1 Fig). Our samples (HKAS45821 and HKAS85517) were closely aligned with the Japanese “*A*. *gallica*” (Fig 2). We propose that *A*. *gallica* is restricted to Europe and the “*A*. *gallica*” in East Asia and North America are not conspecific with the European *A*. *gallica*. Moreover, our phylogenetic analyses indicated that the currently recognized *A*. *gallica* is polyphyletic and probably comprises cryptic species (Fig 2), in accordance with the findings of Klopfenstein et al. [60]. To determine the phylogenetic relationships of these putative *A*. *gallica* collections, seven haplotypes from *tef1*-*α* sequences were detected within 17 samples (S2 Fig), and the phylogenetic tree inferred from the EF1-III dataset, which included these haplotypes, indicated that Lineage 6 could be split into four subgroups, providing preliminary evidence for ongoing speciation (Fig 3).

Most of the newly identified phylogenetic lineages in Clade I and Clade III (Fig 1) are restricted to China. Lineage 1 is distributed from Southwest to Northeast China in broad-leaved forests. The two sympatric phylogenetic lineages, Lineage 2 and Lineage 3, are restricted to Yunnan and Guizhou, Southwest China, and Lineage 5 is found only in Northeast China. In contrast, Lineage 4 is widely distributed in China from the northeastern to the central, southwestern and northwestern parts. Among the nine new phylogenetic lineages identified in this study, Lineage 7 was significantly different from the other species in phylogenetic position. It was formed of a single clade (Clade III) while Lineages 1 to 6 were nested within Clade I (Fig 1).

### *Armillaria* associated with Tianma in China

Prior to this study, six species (including subspecies) of *Armillaria*, namely *A*. *cepistipes*, *A*. *gallica*, *A*. *mellea* subsp. *nipponica*, *A*. *jezoensis* J.Y. Cha & Igarashi, *A*. *tabescens* and *A*. *nabsnona*, had been reported to be associated or form symbiotic relationships with *Gastrodia elata* in Japan [7, 70]. Our study indicated that *A*. *cepistipes* and *A*. *nabsnona* could be associated with Tianma in China (Fig 1). However, we found no evidence for the symbiosis between Tianma and the other four *Armillaria* species based on our data. In contrast, four additional phylogenetic lineages, namely Lineages 2, 3, 6 and Nag. E, were found to be associated with the cultivation of Tianma in China (Fig 1). The collections of Lineage 4 (HKAS85594) and *A*. *mellea* (HKAS85471) were taken from Tianma plantations. However, no direct evidence showed that strains within them could be used for Tianma cultivation. Interestingly, two common features could be found among the *Armillaria* strains suitable for Tianma cultivation: (i) they possibly belonged to saprotrophic/low-pathogenic phylogenetic lineages (Fig 1, Clade I); and (ii) their mycelia had a fast growth speed and could form vigorous and stout rhizomorphs, the branching patterns of which were was monopodial (S3 Fig). Morrison revealed that species producing monopodially branched rhizomorphs, i.e., *A*. *gallica*, *A*. *nabsnona* and *A*. *cepistipes*, were less aggressive than dichotomously branched species (*A*. *mellea*, *A*. *borealis* and *A*. *ostoyae*) [71]. We hypothesize that the low-pathogenic (less virulent and aggressive) character of these species helps *Armillaria* to establish a symbiotic relationship with Tianma, rather than causing disease to tubers, while the rapid growth of mycelia and the healthy development of rhizomorphs greatly improves the ability of *Armillaria* to derive water and nutrition from the soil or rotten wood and share them with the tubers of Tianma.

Two different cultivars of Tianma, “Brown Tianma” (*G*. *elata* f. *glauca*) and “Red Tianma” (*G*. *elata* f. *elata*), are widely cultivated in China [72]. The former is mainly produced in southwest China (Sichuan, northeastern Yunnan and western Guizhou provinces), and the latter is widely cultivated in southwest, central, north and northeast China. Our study revealed that different phylogenetic lineages of *Armillaria* had different cultivation patterns with Tianma. Brown Tianma was associated with strains of *A*. *nabsnona*, *A*. *cepistipes*, Lineage 2, Nag. E, Lineage 3 and Lineage 6, while Red Tianma was only associated with Lineage 6. Our investigation revealed that two strains of Lineage 6 (HKAS86560 and HKAS86558) were widely considered as good promoters for the cultivation of Tianma in central China, and that a few strains belonging to Lineage 2 and Lineage 3 were widely used for the cultivation of Tianma in southwest China (Yunnan, Sichuan and Guizhou provinces). Although the strains of Lineage 6 were introduced into Zhaotong, Yunnan Province to achieve high yields of Brown Tianma, they proved uncompetitive compared with the local strains of *A*. *cepistipes* (HKAS86542), Lineage 2 (HKAS86543) and *A*. *nabsnona* (HKAS86544). These findings strongly suggest that native and/or naturalized *Armillaria* strains should be selected for the cultivation of Tianma, which has also been suggested for the selection of species for agroforestry [73].

## Supporting Information

S1 Fig Phylogenetic trees inferred from the maximum likelihood (ML) analysis with branch support obtained by ML and BI analyses based on ITS, *tef1*-α and *β*-*tubulin* sequences, respectively. Branch support values are indicated by numbers above branches (ML-BP/BI-PP). Thick black branches received high support in the analyses (ML-BP ≥ 70%, BI-PP ≥ 0.95). Taxon labels indicate strain number and geographic source. Branches showing supported conflict with single gene phylogenies are highlighted in different colors.
(PDF)

S2 Fig Best haplotype reconstruction of seven samples with heterozygous sites. (PDF)

S3 Fig The rhizomorphs development of strains associated with Tianma. The strains of a-f were associated with Tianma cultivation and strains of g-h could not be used for Tianma cultivation. a and b: Lineage 6 (HKAS86560, HKAS86558); c: *A*. *nabsnona* (HKAS86544); d: Lineage 2 (HKAS86543); e: *A*. *cepistipes* (HKAS86542); f: Nag. E (HKAS86549); g: Lineage 1 (HKAS85457); h: *A*. *mellea* (HKAS85471).
(PDF)

S1 Table *Tef1*-α sequences from GenBank used in this study and their accession numbers. (DOC)
