# Supplementary figures and images for: Phylogenetic Analyses of Armillaria Reveal at Least 15 Phylogenetic Lineages in China, Seven of Which Are Associated with Cultivated Gastrodia elata
Source: PLoS One. 2016 May 3;11(5):e0154794. doi: 10.1371/journal.pone.0154794 (PMC4854404; doi:10.1371/journal.pone.0154794)

ITS

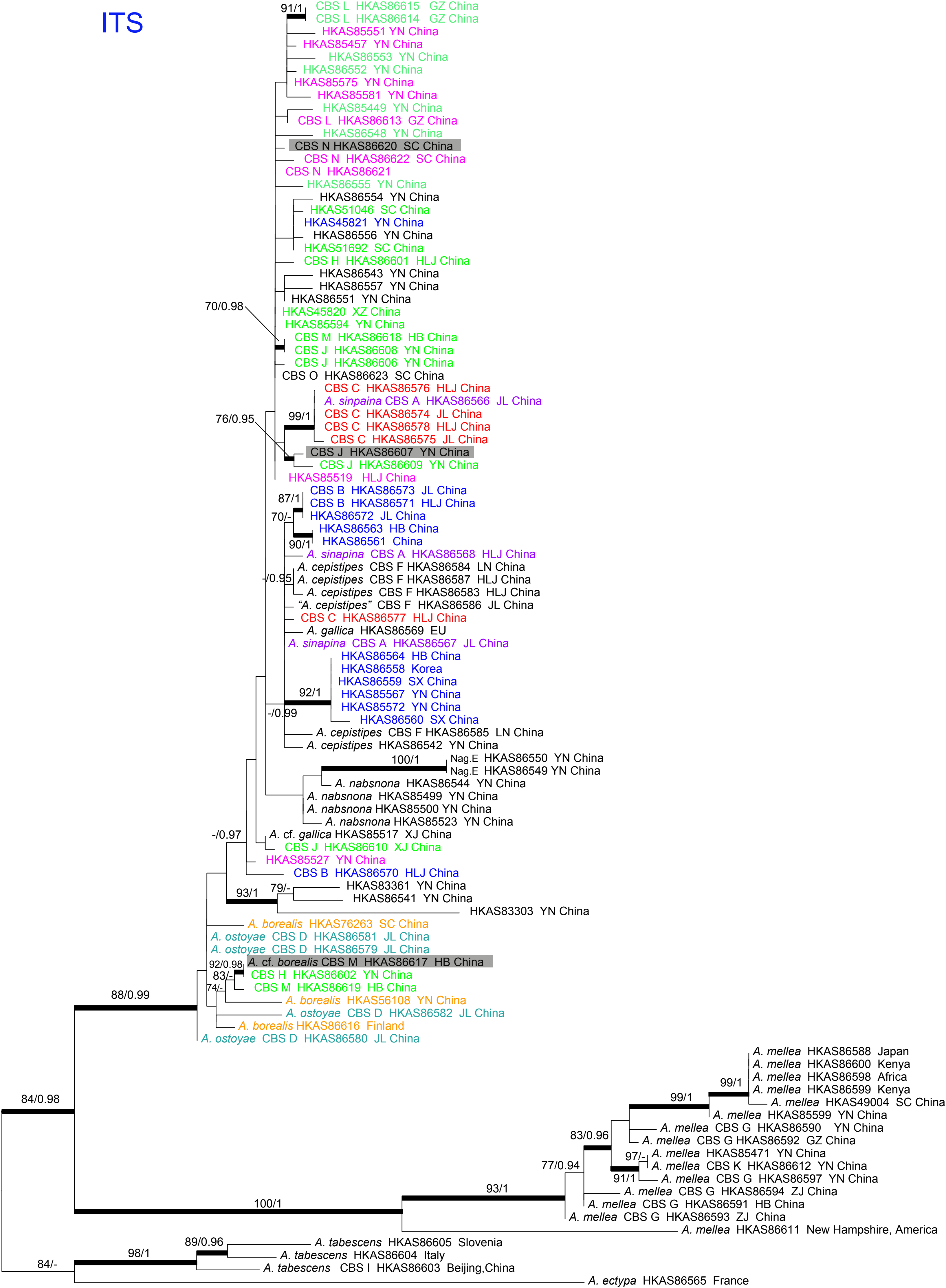

tef1-a

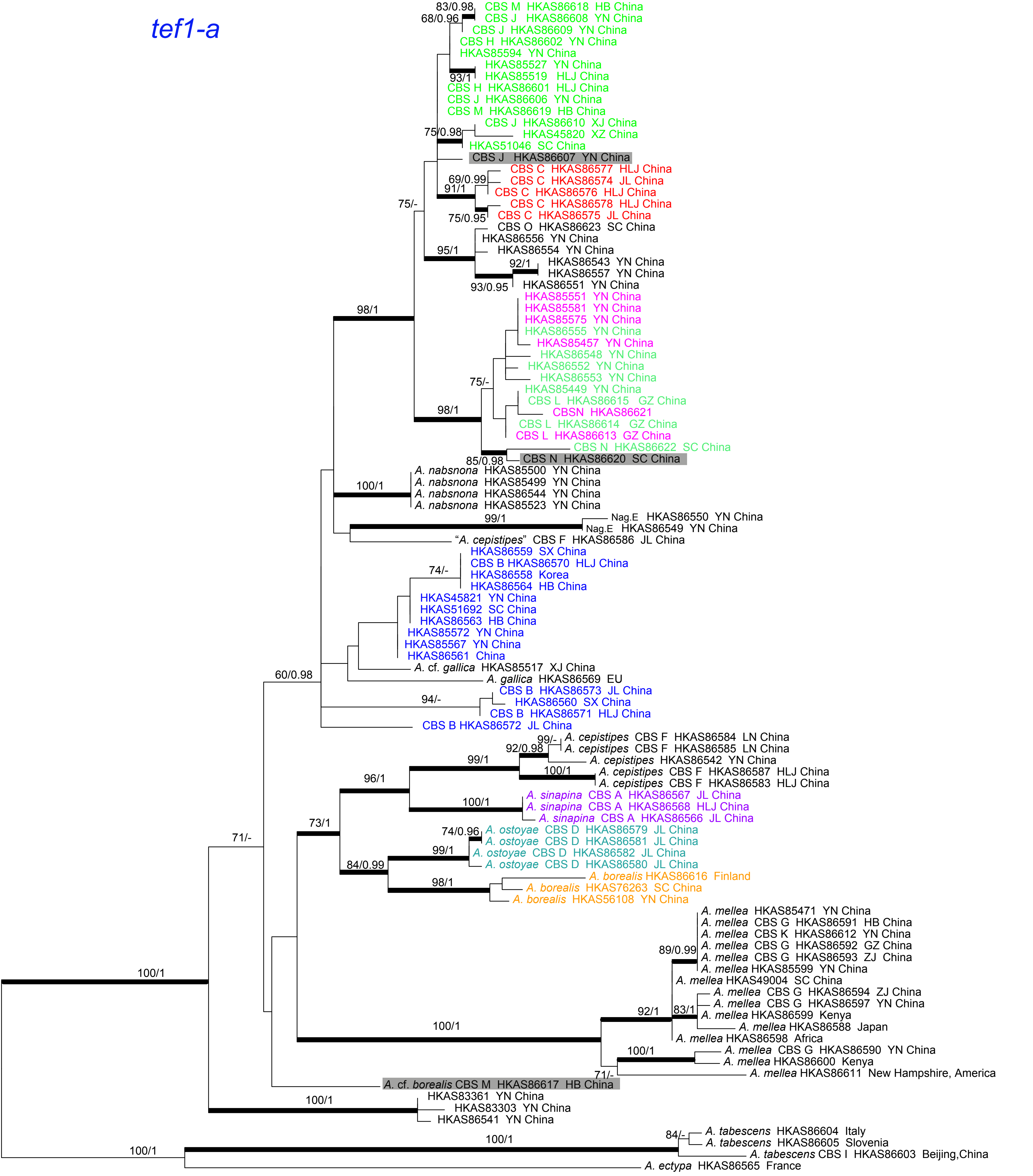

0.03

beta-tubulin

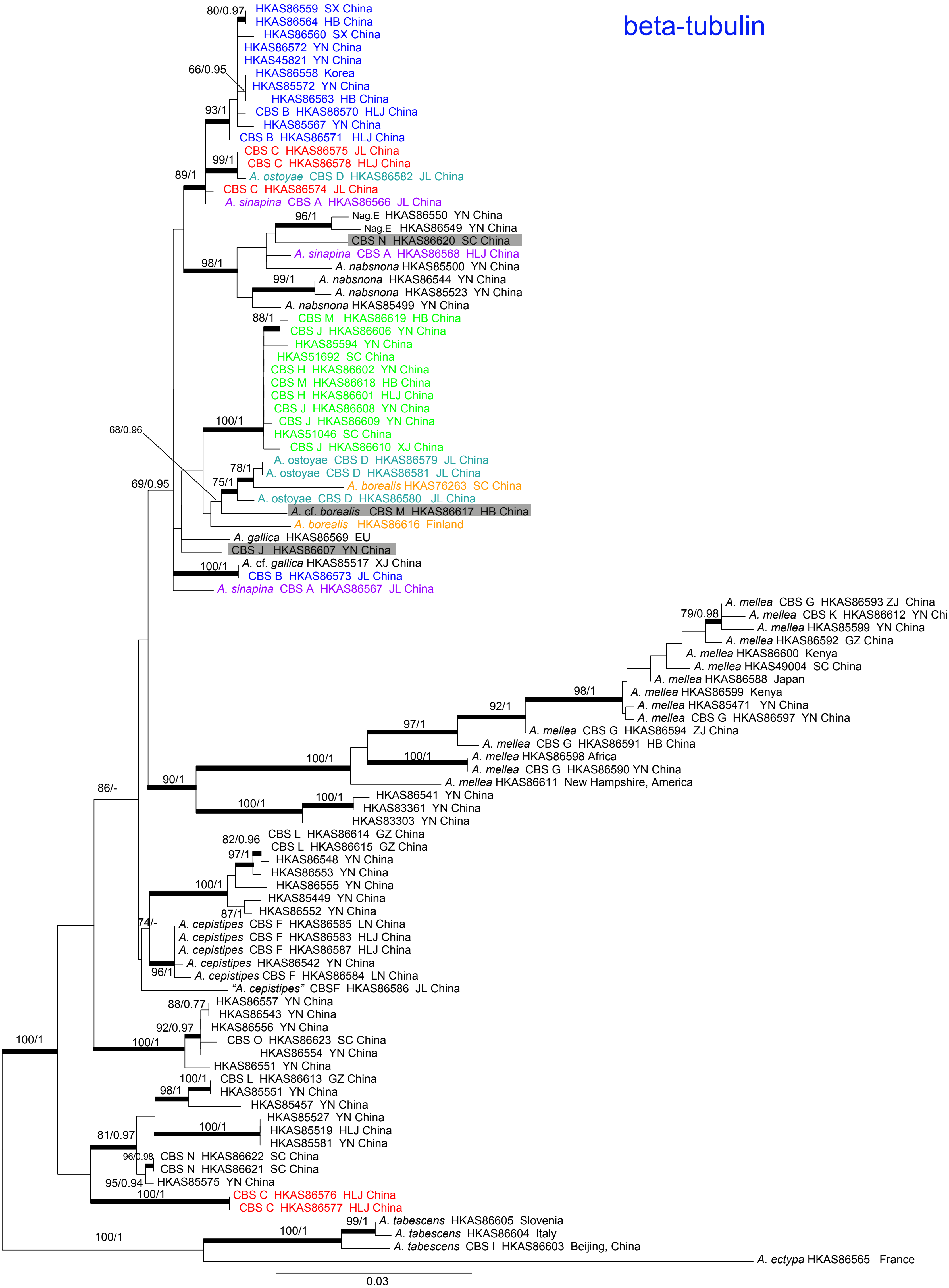

Supplement: S1 Fig — Branch support values are indicated by numbers above branches (ML-BP/BI-PP). Thick black branches received high support in the analyses (ML-BP ≥ 70%, BI-PP ≥ 0.95). Taxon labels indicate strain number and geographic source. Branches showing supported conflict with single gene phylogenies are highlighted in different colors. (PDF) [file pone.0154794.s001.pdf]

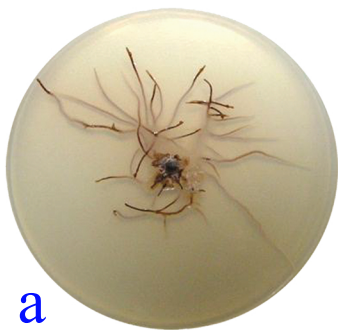

a

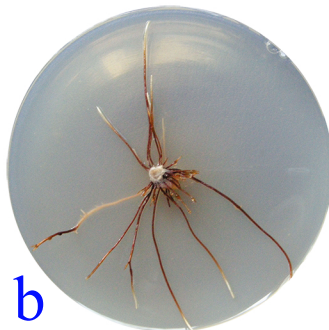

b

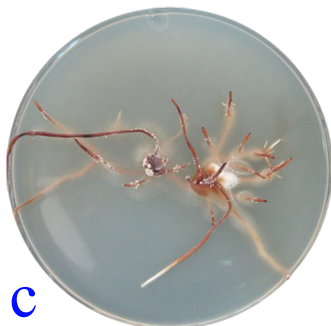

c

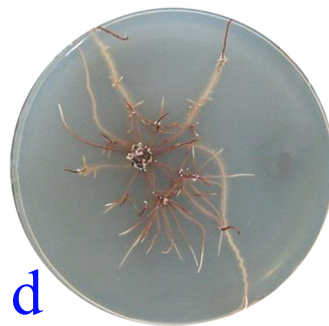

d

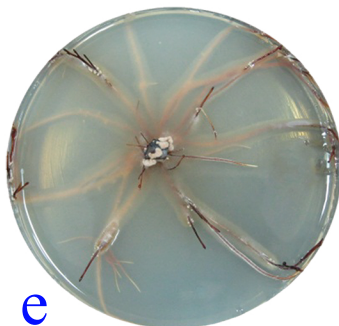

e

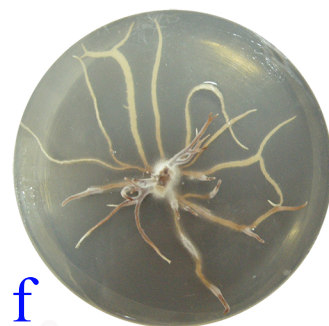

f

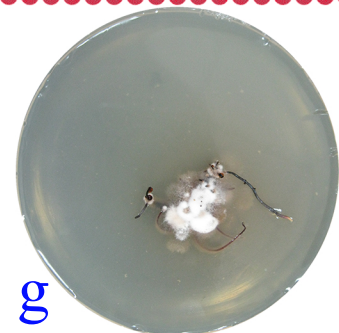

g

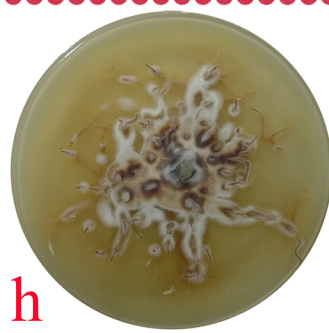

h

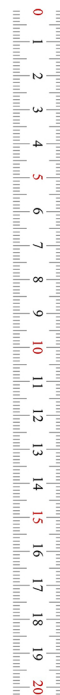

Supplement: S3 Fig — The strains of a-f were associated with Tianma cultivation and strains of g-h could not be used for Tianma cultivation. a and b: Lineage 6 (HKAS86560, HKAS86558); c: A. nabsnona (HKAS86544); d: Lineage 2 (HKAS86543); e: A. cepistipes (HKAS86542); f: Nag. E (HKAS86549); g: Lineage 1 (HKAS85457); h: A. mellea (HKAS85471). (PDF) [file pone.0154794.s003.pdf]
