# Supplementary material for: Phylogenetic Analyses of Armillaria Reveal at Least 15 Phylogenetic Lineages in China, Seven of Which Are Associated with Cultivated Gastrodia elata
Source: PLoS One. 2016 May 3;11(5):e0154794. doi: 10.1371/journal.pone.0154794 (PMC4854404; doi:10.1371/journal.pone.0154794)
Supplement: S2 Fig — (PDF) [file pone.0154794.s002.pdf]

	018	036	051	096	130	143	144	162	163	164	183	204	207	216	219	285	288	291	318	328	330	331	346	347	360	396	405	435	438	447
H1	A	C	G	C	G	A	C	C	T	A	C	T	C	T	C	C	T	T	A	C	A	C	T	T	C	G	C	T	C	C
H2	A	C	A	T	G	G	C	T	C	A	C	T	C	C	C	C	T	C	G	C	G	C	C	C	T	G	C	C	T	C
H3	A	C	A	T	G	G	C	T	C	A	C	T	C	C	C	T	T	C	G	C	G	C	C	C	T	G	C	C	T	C
H4	G	C	G	C	G	G	T	C	T	G	C	C	C	C	T	C	T	T	A	C	A	C	T	T	C	G	C	T	C	C
H5	G	C	G	C	G	G	T	C	T	G	T	C	C	C	T	C	T	T	A	C	A	C	T	T	C	G	T	T	C	T
H6	G	C	G	C	A	G	T	C	T	G	C	C	C	C	T	C	T	T	A	C	A	C	T	T	C	G	C	T	C	C
H7	G	T	G	C	G	G	C	C	T	G	C	C	T	C	C	C	C	T	A	T	A	A	C	C	C	A	C	T	C	C

S2 Fig Sequence variations of seven haplotypes.

Species or phylogenetic lineages	Sample	Heterogeneous sites	Haplotype (H)
A. cf. *gallica*[R]	HKAS85517	0	H1
A. gallica[R]	NA13	0	H1
A. gallica[R]	2000-46	0	H1
Lineage 6[R]	HKAS86570[B]	0	H5
Lineage 6[R]	HKAS86571[B]	0	H2
Lineage 6[R]	HKAS86572[B]	0	H7
Lineage 6[R]	HKAS86573[B]	0	H2
Lineage 6[R]	HKAS86560	0	H3
Lineage 6[R]	HKAS86561	0	H4
Lineage 6[R]	HKAS86563	0	H4
Lineage 4	HKAS51692	8	H1, H6
Lineage 6	HKAS45821	8	H1, H6
Lineage 6	HKAS85567	20	H2, H5
Lineage 6	HKAS85572	20	H2, H5
Lineage 6	HKAS86558	20	H2, H5
Lineage 6	HKAS86559	20	H2, H5
Lineage 6	HKAS86564	20	H2, H5

[R] The homozygous individuals in the table were used as reference to calculate the best haplotypes for the seven heterozygous isolates.

[B] The strain was originally identified as CBS B (*A. gallica*) based on mating tests by other authors but did not match the phylogenetic lineage identification in the current study.
