# Supplementary material for: Phylogenetic Analyses of Armillaria Reveal at Least 15 Phylogenetic Lineages in China, Seven of Which Are Associated with Cultivated Gastrodia elata
Source: PLoS One. 2016 May 3;11(5):e0154794. doi: 10.1371/journal.pone.0154794 (PMC4854404; doi:10.1371/journal.pone.0154794)
Supplement: S1 Table — (DOC) [file pone.0154794.s004.doc]

**S1 Table.** **The translation elongation factor-1 alpha** **(*tef1-a*) sequences from GenBank used in this study and their accession numbers.**

| **Species** | **Original identification** | **Distribution** | **Specimen** | **Fungal**  **source** | **Accession**  **number** | **Reference** |
| --- | --- | --- | --- | --- | --- | --- |
| *A. altimontana* | Mating tests, sequence analysis of *tef1-a* | Idaho, USA | POR100 | single spore | JN944606 |  |
| *A. altimontana* | Mating tests, sequence analysis of *tef1-a* | Idaho, USA | Mac6 | basidioma | JN944607 |  |
| *A. altimontana* | Mating tests, sequence analysis of *tef1-a* | Idaho, USA | D84 | single spore | JN944609 |  |
| *A.* *borealis* | Mating tests, sequence analysis of IGS, ITS and *tef1-a* | Finland | A2 | single spore | HQ285901 |  |
| *A. borealis* | Mating tests, sequence analysis of IGS, ITS and *tef1-a* | Finland | A1 | single spore | JN657494 |  |
| *A. borealis* | Mating tests, sequence analysis of IGS, ITS and *tef1-a* | Germany | A5 | single spore | JN657495 |  |
| *A. borealis* | Mating tests, sequence analysis of IGS, ITS and *tef1-a* | Switzerland | A618 | Rhizomorph | JN657496 |  |
| *A. borealis* | Mating tests, sequence analysis of IGS, ITS and *tef1-a* | Switzerland | A722 | Rhizomorph | JN657497 |  |
| *A. calvescens* | Mating tests, sequence analysis of *tef1-a*, *rpb2* and nLSU | Michigan, USA | ST17 | single-spore | JF313130 |  |
| *A. calvescens* | Mating tests, sequence analysis of *tef1-a*, *rpb2* and nLSU | Quebec, USA | ST3 | single-spore | JF313138 |  |
| *A. calvescens* | Mating tests, sequence analysis of *tef1-a*, *rpb2* and nLSU | Ontario, USA | Ac98 | single-spore | JF895833 |  |
| *A. cepistipes* | Mating tests, sequence analysis of IGS, ITS and *tef1-a* | Aomori, Japan | ND11 | single spore | AB510787 |  |
| *A. cepistipes* | Mating tests, sequence analysis of IGS, ITS and *tef1-a* | Yamagata, Japan | 94-33-01 | single spore | AB510789 |  |
| *A. cepistipes* | Mating tests, sequence analysis of *tef1-a* | Yamagata, Japan | 94-31 | single spore | AB539497 |  |
| *A. cepistipes* | Mating tests, sequence analysis of *tef1-a* | Gunma, Japan | 92-19 | single spore | AB539498 |  |
| *A. cepistipes* | Mating tests, sequence analysis of *tef1-a α* | Tokyo, Japan | A-14 | single spore | AB539499 |  |
| *A. cepistipes* | Mating tests, sequence analysis of *tef1-a* | Ishikawa,Japan | 01-11 | single spore | AB539505 |  |
| *A. cepistipes* | Mating tests, sequence analysis of IGS, ITS and *tef1-a* | Washington, USA | W113 | basidioma | JF313115 |  |
| *A. cepistipes* | Mating tests, sequence analysis of IGS, ITS and *tef1-a* | British Columbia, Canda | M110 | basidioma | JF313121 |  |
| *A. cepistipes* | Mating tests, sequence analysis of *tef1-a* | Tampere, Finland | EB2 | single spore | JF746911 |  |
| *A. cepistipes* | Mating tests, sequence analysis of *tef1-a* | Finland | EB3 | single spore | JF746912 |  |
| *A. cepistipes* | Mating tests, sequence analysis of nLSU-IGS and *tef1-a* | Italy | B5 | single spore | KJ414321 |  |
| *A. ectypa* | Sequence analysis of ITS and *tef1-a* | Aomori, Japan | Je-4 | basidioma | AB558993 |  |
| *A. ectypa* | Sequence analysis of ITS and *tef1-a* | Aomori, Japan | Je-9 | basidioma | AB558995 |  |
| *A. ectypa* | Morphology, sequence analysis of IGS, ITS and *tef1-a* | Austria | BRNM704974 | basidioma | EU251403 |  |
| *A. gallica* | Mating tests, sequence analysis of IGS, ITS and *tef1-a* | Fukushima,Japan | NA13 | single spore | AB510760 |  |
| *A. gallica* | Mating tests, sequence analysis of *tef1-a* | Japan | 2000-46 | single spore | AB539483 |  |
| *A. gallica* | Mating tests, sequence analysis of IGS, ITS and *tef1-a* | British Columbia, Canada | M70 | basidioma | JF313123 |  |
| *A. gallica* | Mating tests, sequence analysis of IGS, ITS and *tef1-a* | Michigan, USA | ST22 | basidioma | JF313126 |  |
| *A. gallica* | Mating tests, sequence analysis of *tef1-a* | France | EE4 | single spore | JF746919 |  |
| *A. gallica* | Mating tests, sequence analysis of *tef1-a* | France | EE5 | single spore | JF746920 |  |
| *A. gallica* | Mating tests, sequence analysis of *tef1-a*, *rpb2* and nLSU | Ontario, USA | Aga81 | single spore | JF895846 |  |
| *A. gallica* | Mating tests, sequence analysis of *tef1-a*, *rpb2* and nLSU | Ontario, USA | Aga235 | single spore | JF895847 |  |
| *A. gallica* | Mating tests, sequence analysis of *tef1-a*, *rpb2* and nLSU | Wisconsin, USA | ST23 | single spore | JF895849 |  |
| *A. gallica* | Mating tests, sequence analysis of nLSU-IGS and *tef1-a* | Germany | 84-087 | single spore | KJ200950 |  |
| *A. gallica* | Mating tests, sequence analysis of nLSU-IGS and *tef1-a* | Dole, France | 84-088 | single spore | KJ200953 |  |
| *A. gallica* | Mating tests, sequence analysis of nLSU-IGS and *tef1-a* | Iran | 86-008 | single spore | KJ200954 |  |
| *A. gallica* | Mating tests, sequence analysis of nLSU-IGS and *tef1-a* | Montlucon, France | 86-032 | single spore | KJ200955 |  |
| *A. gemina* | Mating tests, sequence analysis of *tef1-a* | West Virginia, USA | ST11 | unknown | JF313133 |  |
| *A. gemina* | Mating tests, sequence analysis of *tef1-a* | New York, USA | ST9 | basidioma | JF313135 |  |
| *A. gemina* | Mating tests, sequence analysis of *tef1-a* | New York, USA | ST8 | basidioma | JF313136 |  |
| *A. mellea* | Mating tests, sequence analysis of IGS, ITS and *tef1-a* | Tokyo, Japan | A-10 | mycerial mat | AB510800 |  |
| *A. mellea* | Mating tests, sequence analysis of *tef1-a* | Oita, Japan | AS-1 | no record | AB539508 |  |
| *A. mellea* | Mating tests, sequence analysis of *tef1-a* | Yamanashi, Japan | 92-41 | mycelial mat | AB539510 |  |
| *A. mellea* | Mating tests, sequence analysis of *tef1-a* | Fukushima, Japan | 97-29 | basidioma | AB539512 |  |
| *A. mellea* | Mating tests, sequence analysis of *tef1-a* | New Hampshire, USA | ST21 | multisporous | JF313127 |  |
| *A. mellea* | Mating tests, sequence analysis of *tef1-a* | Wisconsin, USA | ST20 | basidioma | JF313128 |  |
| *A. mellea* | Mating tests, sequence analysis of *tef1-a* | Virginia, USA | ST5 | multisporous | JF313137 |  |
| *A. mellea* | Mating tests, sequence analysis of nLSU, *rpb2* and *tef1-a* | Ontario, USA | Am115 | mycelial fans | JF895857 |  |
| *A. mellea* | Mating tests, sequence analysis of *rpb2* and *tef1-a* et al. | Surrey, England | FP-135350-Sp | basidioma | JN796025 |  |
| *A. mellea* | Mating tests, sequence analysis of *rpb2* and *tef1-a* et al. | Madeira, Portugal | 97081/1 | single spore | JN796028 |  |
| *A. mellea* | Mating tests, sequence analysis *rpb2* and *tef1-a* et al. | Trentino, Italy | 03277/2 | single spore | JN796036 |  |
| *A. nabsnona* | Mating tests, sequence analysis of IGS, ITS and *tef1-a* | Tottori, Japan | NB3 | single spore | AB510763 |  |
| *A. nabsnona* | Mating tests, sequence analysis of IGS, ITS and *tef1-a* | Ibaraki, Japan | 00-16-4 | single spore | AB510765 |  |
| *A. nabsnona* | Mating tests, sequence analysis of IGS, ITS and *tef1-a* | Aomori, Japan | 00-3-1 | single spore | AB510766 |  |
| *A. nabsnona* | Mating tests, sequence analysis of IGS, ITS and *tef1-a* | Idaho, USA | C21 | basidioma | JF313119 |  |
| *A. nabsnona* | Mating tests, sequence analysis of IGS, ITS and *tef1-a* | British Columbia, Canada | M90 | basidioma | JF313122 |  |
| *A. nabsnona* | Mating tests, sequence analysis of IGS, ITS and *tef1-a* | Alaska, USA | ST16 | multisporous | JF313124 |  |
| *A. ostoyae* | Mating tests, sequence analysis of *tef1-a* | Mie, Japan | 00-10 | basidioma | AB539463 |  |
| *A. ostoyae* | Mating tests, sequence analysis of *tef1-a* | Iwate, Japan | 94-72 | basidioma | AB539469 |  |
| *A. ostoyae* | Mating tests, sequence analysis of *tef1-a* | Hokkaido, Japan | 05-82 | basidioma | AB539479 |  |
| *A. ostoyae* | Mating tests, sequence analysis of *tef1-a* | Nurmijarn, Finland | EC4 | single spore | JF746925 |  |
| *A. ostoyae* | Mating tests, sequence analysis of *tef1-a* | France | EC5 | basidioma | JF746926 |  |
| *A. sinapina* | Mating tests, sequence analysis of IGS, ITS and *tef1-a* | Hokkaido, Japan | 96-7-1 | single spore | AB510774 |  |
| *A. sinapina* | Mating tests, sequence analysis of *tef1-a* | Nagano, Japan | 90-07 | basidioma | AB539492 |  |
| *A. sinapina* | Mating tests, sequence analysis of *tef1-a* | Yamanashi, Japan | 2002-65 | rhizomorph | AB539493 |  |
| *A. sinapina* | Mating tests, sequence analysis of *tef1-a* | Hokkaido, Japan | 05-21 | no record | AB539494 |  |
| *A. sinapina* | Mating tests, sequence analysis of IGS, ITS and *tef1-a* | British Columbia, Canada | M50 | basidioma | JF313114 |  |
| *A. sinapina* | Mating tests, sequence analysis of IGS, ITS and *tef1-a* | Michigan, USA | ST13 | multisporous | JF313131 |  |
| *A. sinapina* | Mating tests, sequence analysis of IGS, ITS and *tef1-a* | Washington, USA | ST12 | basidioma | JF313132 |  |
| *A. solidipes* | Mating tests, sequence analysis of IGS, ITS and *tef1-a* | Washington, USA | ST2 | basidioma | JF313139 |  |
| *A. solidipes* | Mating tests, sequence analysis of IGS, ITS and *tef1-a* | Idaho, USA | P1404 | basidioma | JF313140 |  |
| *A. solidipes* | Mating tests, sequence analysis of IGS, ITS and *tef1-a* | New Hampshire, USA | ST1 | multisporous | JF313141 |  |
| *A. tabescens* | Mating tests, sequence analysis of IGS, ITS and *tef1-a* | Ibaraki, Japan | 96-1-8 | single spore | AB510804 |  |
| *A. tabescens* | Mating tests, sequence analysis of IGS, ITS and *tef1-a* | Gunma, Japan | 2006-20-01 | single spore | AB510807 |  |
| *A. tabescens* | Mating tests, sequence analysis of *tef1-a* | Ibaraki, Japan | 02-26 | mycelial mat | AB539514 |  |
| *A. tabescens* | Mating tests, sequence analysis of IGS, ITS and *tef1-a* | USA | OOI99 | basidioma | JF313112 |  |
| *A. tabescens* | Mating tests, sequence analysis of *tef1-a* | Puy de Dome, France | ET3 | single spore | JF746929 |  |
| Nag. E | Mating tests, sequence analysis of IGS, ITS and *tef1-a* | Kanagawa, Japan | 96-37-1 | single spore | AB510769 |  |
| Nag. E | Mating tests, sequence analysis of IGS, ITS and *tef1-a* | Tottori, Japan | NE4 | single spore | AB510771 |  |
| Nag. E | Mating tests, sequence analysis of IGS, ITS and *tef1-a* | Aomori, Japan | 2000-23-02 | single spore | AB510773 |  |

1. Brazee NJ, Ortiz-Santana B, Banik MT, Lindner DL. *Armillaria altimontana*, a new species from the western interior of North America. Mycologia. 2012;104(5):1200-1205.

2. Tsykun T, Rigling D, Prospero S. A new multilocus approach for a reliable DNA-based identification of *Armillaria* species. Mycologia. 2013;105(4):1059-1076. doi: 10.3852/12-209. PubMed PMID: 23449075.

3. Antonín V, Tomšovský M, Sedlák P, Májek T, Jankovský L. Morphological and molecular characterization of the *Armillaria cepistipes*-*A*. *gallica* complex in the Czech Republic and Slovakia. Mycol Prog. 2009;8(3):259-271. doi: 10.1007/s11557-009-0597-1.

4. Ross-Davis AL, Hanna JW, Klopfenstein NB, Kim MS. Advances toward DNA-based identification and phylogeny of North American *Armillaria* species using elongation factor-1 alpha gene. Mycoscience. 2012;53(2):161-165. doi: 10.1007/s10267-011-0148-x.

5. Hasegawa E, Ota Y, Hattori T, Kikuchi T. Sequence-based identification of Japanese *Armillaria* species using the elongation factor-1 alpha gene. Mycologia. 2010;102(4):898-910. doi: 10.3852/09-238.

6. Hasegawa E, Ota Y, Hattori T, Sahashi N, Kikuchi T. Ecology of Armillaria species on conifers in Japan. Forest Pathol. 2011;41(6):429-437. doi: 10.1111/j.1439-0329.2010.00696.x.

7. Mulholland V, MacAskill GA, Laue BE, Steele H, Kenyon D, Green S. Development and verification of a diagnostic assay based on EF-1 α for the identification of *Armillaria* species in Northern Europe. Forest Pathol. 2012;42(3):229-238. doi: 10.1111/j.1439-0329.2011.00747.x.

8. Keca N, Klopfenstein N, Kim M, Solheim H, Woodward S. Initial characterization of an unidentified *Armillaria* isolate from Serbia using LSU-IGS1 and TEF-1-a genes. 2014.

9. Ota Y, Kim M-S, Neda H, Klopfenstein NB, Hasegawa E. The phylogenetic position of an Armillaria species from Amami-Oshima, a subtropical island of Japan, based on elongation factor and ITS sequences. Mycoscience. 2011;52(1):53-58. doi: 10.1007/s10267-010-0066-3.

10. Brazee NJ, Hulvey JP, Wick RL. Evaluation of partial tef1, rpb2, and nLSU sequences for identification of isolates representing Armillaria calvescens and Armillaria gallica from northeastern North America. Fungal biology. 2011;115(8):741-749. doi: 10.1016/j.funbio.2011.05.008. PubMed PMID: 21802054.

11. Baumgartner K, Baker BR, Korhonen K, Zhao J, Hughes KW, Bruhn J, et al. Evidence of natural hybridization among homothallic members of the basidiomycete *Armillaria mellea sensu stricto*. Fungal biology. 2012;116(6):677-691. doi: 10.1016/j.funbio.2012.03.006. PubMed PMID: 22658313.
